# Identification of Ice Plant (*Mesembryanthemum crystallinum* L.) MicroRNAs Using RNA-Seq and Their Putative Roles in High Salinity Responses in Seedlings

**DOI:** 10.3389/fpls.2016.01143

**Published:** 2016-08-09

**Authors:** Chih-Pin Chiang, Won C. Yim, Ying-Hsuan Sun, Miwa Ohnishi, Tetsuro Mimura, John C. Cushman, Hungchen E. Yen

**Affiliations:** ^1^Department of Life Sciences, National Chung Hsing UniversityTaichung, Taiwan; ^2^Department of Biochemistry and Molecular Biology, University of NevadaReno, NV, USA; ^3^Department of Forestry, National Chung Hsing UniversityTaichung, Taiwan; ^4^Graduate School of Science, Kobe UniversityKobe, Japan

**Keywords:** halophyte, ice plant, miRNA, root growth, salinity responses, small RNA profile

## Abstract

The halophyte *Mesembryanthemum crystallinum* (common or crystalline ice plant) is a useful model for studying molecular mechanisms of salt tolerance. The morphology, physiology, metabolism, and gene expression of ice plant have been studied and large-scale analyses of gene expression profiling have drawn an outline of salt tolerance in ice plant. A rapid root growth to a sudden increase in salinity was observed in ice plant seedlings. Using a fluorescent dye to detect Na^+^, we found that ice plant roots respond to an increased flux of Na^+^ by either secreting or storing Na^+^ in specialized cells. High-throughput sequencing was used to identify small RNA profiles in 3-day-old seedlings treated with or without 200 mM NaCl. In total, 135 conserved miRNAs belonging to 21 families were found. The hairpin precursor of 19 conserved mcr-miRNAs and 12 novel mcr-miRNAs were identified. After 6 h of salt stress, the expression of most mcr-miRNAs showed decreased relative abundance, whereas the expression of their corresponding target genes showed increased mRNA relative abundance. The cognate target genes are involved in a broad range of biological processes: transcription factors that regulate growth and development, enzymes that catalyze miRNA biogenesis for the most conserved mcr-miRNA, and proteins that are involved in ion homeostasis and drought-stress responses for some novel mcr-miRNAs. Analyses of the functions of target genes revealed that cellular processes, including growth and development, metabolism, and ion transport activity are likely to be enhanced in roots under salt stress. The expression of eleven conserved miRNAs and two novel miRNAs were correlated reciprocally with predicted targets within hours after salt stress exposure. Several conserved miRNAs have been known to regulate root elongation, root apical meristem activity, and lateral root formation. Based upon the expression pattern of miRNA and target genes in combination with the observation of Na^+^ distribution, ice plant likely responds to increased salinity by using Na^+^ as an osmoticum for cell expansion and guard cell opening. Excessive Na^+^ could either be secreted through the root epidermis or stored in specialized leaf epidermal cells. These responses are regulated in part at the miRNA-mediated post-transcriptional level.

## Introduction

*Mesembryanthemum crystallinum* is a halophyte that can grow in high saline soils that have levels of sodium equivalent to that found in sea water. As an ecological adaptation to Mediterranean climates, ice plant increases its salt-tolerant ability during development (Adams et al., 1998). To date, the best-studied response is the progressive development of crassulacean acid metabolism (CAM) at the juvenile-adult transition stage (Cushman, 2001) brought about by water-deficit stress arising from low relative humidity or saline conditions encountered in the environment (Winter and Holtum, 2007). At the seedling stage, ice plant already exhibits moderate salt tolerance, and in fact, upon germination, ice plant seedlings are able to tolerate saline soils containing 150 mM NaCl, a condition that inhibits growth of glycophytes (Bohnert and Cushman, 2000). As growth proceeds, water and salt ions are stored in epidermal bladder cells (EBCs), specialized cells that cover the aerial part of plant and boost salt tolerance (Agarie et al., 2007). The salt-tolerant mechanism of ice plant is regulated at the chromatin, transcriptional, post-transcriptional, translational, and post-translational levels. For example, gene coding for the key CAM cycle enzyme phosphoenolpyruvate carboxylase was found to be regulated at the level of DNA methylation (Huang et al., 2010), transcription activation, post-transcriptional regulation (Cushman et al., 1989), and protein phosphorylation (Nimmo, 2000). Although the genome of ice plant has not yet been completely resolved, large-scale gene expression profiling studies have shown salt-induced changes of thousands of genes using the conventional expressed sequence tag analysis (Kore-eda et al., 2004), an oligonucleotide microarray (Cushman et al., 2008), and next-generation sequencing (NGS; Oh et al., 2015; Tsukagoshi et al., 2015). A large number of genes that function in ion transport, metabolism, osmolyte accumulation, and energy generation are regulated under salt stress in temporal-, stage-, or tissue-specific manners.

MiRNAs are small endogenous non-coding RNAs conserved in eukaryotic cells. They regulate gene expression post-transcriptionally by binding to mRNA, thereby either degrading the target mRNA directly or repressing its translation (Bartel, 2004; Vazquez, 2006). In plants, primary transcripts of miRNA (pri-miRNA) are transcribed by RNA polymerase II, capped, and polyadenylated (Chen, 2005). The pri-miRNA is then processed through Dicer-like 1 (DCL1) cleavage to form a 20–22 nt miRNA duplex (Kurihara and Watanabe, 2004). The duplex is 2′ O-methylated at the 3′ end by HUA ENHANCER1 and incorporated into an RNA-induced silencing complex, which carries specific miRNAs that recognize the complementary sequence on the target gene and regulates its expression (Li et al., 2005; Yu et al., 2005).

The role of miRNAs in higher plants has been studied extensively. Many miRNA targets are transcription factors and are involved in various stages of development, including organ development, cell differentiation, leaf morphogenesis, floral transition, and seed germination (Jin et al., 2013; Wu, 2013). In addition to the roles of plant miRNA in the regulation of growth and development, some miRNAs are also involved in plant responses to abiotic stress, including light, temperature, water status, and nutrient deficiency (Sunkar et al., 2012). For example, miR398 targets two closely related Cu/Zn superoxide dismutases, CSD1 and CSD2 (Sunkar et al., 2006), as well as a copper chaperone for superoxide dismutase CCS1 (Beauclair et al., 2010). Under oxidative stress, the relative expression of miR398 is decreased, which leads to the post-transcriptional increase of CSD1, CSD2, and CCS1 expression to eliminate reactive oxygen species. When cells encounter an environment deficient in copper, miR398 expression is induced, leading to decreased CSD1, CSD2, and CCS1 expression, causing a release of copper to build essential proteins, such as mitochondrial respiratory complex IV (Chu et al., 2005). MiR399 regulates phosphate homeostasis by targeting *PHO2*, which encodes for a ubiquitin-conjugating enzyme (Chiou et al., 2006). PHO2 is involved in the ubiquitin-mediated protein degradation of phosphate transporter, PHO1, which contributes to loading phosphate into the xylem. During phosphate starvation, the expression of miR399 is increased and the expression of *PHO2* decreases. As a result, PHO1 accumulates in the endomembrane and facilitates phosphate uptake in the roots (Liu et al., 2012). The targets of stress-related miRNA appear to be integral to stress responses and provide a novel platform for understanding plant development and abiotic stress.

Salt tolerance is a complicated process that involves coordination of all parts of plant, from cells to tissues to organs and even entire plants. The salt-tolerant mechanism is regulated at both the transcriptional and post-transcriptional levels. Many transcription factors were demonstrated to be involved in salt-tolerant mechanisms during salt stress (Seki et al., 2002; Golldack et al., 2011). At the level of post-transcriptional control, changes in miRNA expression profiles in response to salt stress were identified in the model plants *Arabidopsis thaliana* (Liu et al., 2008), *Oryza sativa* (Sunkar et al., 2008), and *Zea mays* (Ding et al., 2009). A few studies have identified miRNAs from halophytes, including *Thellungiela salsuginea* (Zhang et al., 2013), *Salicornia brachiata* (Singh and Jha, 2014), *Salicornia europaea* (Feng et al., 2015), and *Avicennia marina* (Khraiwesh et al., 2013). Although genome and transcriptome information is relatively limited in halophytes, highly conserved miRNAs are found to regulate certain transcription factors in halophytes. Some salt-responsive miRNAs function in stress responses and signaling, protein turnover, and ion homeostasis (Feng et al., 2015). These findings extend the idea of miRNAs as ubiquitous regulators under salt-stress conditions in halophytes.

In this report, small RNA profiles were analyzed using RNA-Seq in ice plant seedlings under salt stress. Conserved and novel ice plant miRNAs were identified, target genes for miRNAs were predicted, and expression patterns of selected miRNAs and their potential targets were examined by RT-qPCR. Based upon the expression profiles of selected miRNAs and their targets, the involvement of miRNA-mediated post-transcriptional regulation in salt tolerance is discussed.

## Materials and methods

### Plant materials

Sterile ice plant (*Mesembryanthemum crystallinum* L.) seeds were sown in 1X Murashige and Skoog basal medium with vitamins (*Phyto*Technology Laboratories) and maintained in a vertical position under conditions of 16 h 100 μmol m^−2^s^−1^ light/8 h dark at 25°C (light grown) or continuous darkness at 25°C (dark grown). A vertical position allows roots can be treated by salt. During the salt treatment time, agar plates were placed at a slight angle and control (liquid MS medium) or salt (MS plus 200 mM NaCl) were added directly to plates to cover the lower part of the roots, but not the upper part of seedlings. During treatment period, the solution was gently mixed occasionally to ensure even distribution of NaCl. At the end of treatment, seedlings were washed briefly in distilled water, blotted dry, and then RNA extraction or tissue staining was conducted. Seeds used in this study were collected from plants treated with 350 mM NaCl for 3 weeks during CAM transition stage and Na^+^ content was determined according to Ho et al. (2010).

### Fluorescent detection of Na^+^ distribution

Roots of light-grown 1 and 2-week-old seedlings were immersed in 1% sucrose (control) or 1% sucrose with 200 mM NaCl (salt) for 6 or 24 h. Fresh intact roots and leaf slices (100–200 μm) were stained with 10 μM Sodium Green™ tetraacetate (Invitrogen™ Thermo-Fisher Scientific, Inc.) for 30 min. After washing with MS medium three times, samples were mounted directly on microscopic slides and observed by laser scanning confocal microscopy (Olympus FV-1000). One- and two-week-old seedlings were used in these experiments because these conditions were optimal to document subcellular changes in Na^+^ distribution within the roots, cotyledons, and first leaf pair.

### RNA extraction and high-throughput sequencing

Three-day-old dark-grown ice plant seedlings were immersed in water (control) or 200 mM NaCl (salt) for 6 h. These conditions were selected because it was possible to obtain high-quality, active primary roots. Furthermore, previous studies had shown that under these conditions salt-induced changes in subcellular localization of proteins could be documented (Chiang et al., 2013). Total RNA was extracted using TRIzol® (Invitrogen) according to the manufacturer's instructions. RNA-Seq libraries of control and salt treatment were prepared from total RNA using poly (A)^+^ RNA- or small RNA-enriched samples. Small RNA fractions of 15–30 nt were purified using 15% denaturing acrylamide gel. The sequences of 4 libraries were obtained using the Illumina Hiseq 2000 platform (Illumina, San Diego, CA) at YourGene Bioscience, Taiwan. The datasets of small RNA libraries were deposited to NCBI under GEO accession GSE83508.

### Identification of conserved and novel miRNAs

Analysis of small-RNA reads was based on Motameny et al. (2010). Raw sequences of small RNAs from control and salt treated seedlings were parsed by removing the adaptor sequences, sequence lengths outside 18–25 nt, sequences containing ambiguous nucleotides, and t/r RNA contamination. The filtered unique reads were compared to known mature plant miRNA deposited in the miRBase database (http://www.mirbase.org; Kozomara and Griffiths-Jones, 2014) using the miRProf tool (UEA sRNA toolkit; http://srna-workbench.cmp.uea.ac.uk; Stocks et al., 2012) with no more than two mismatches allowed. To identify the conserved and novel miRNAs in ice plant, unique sequences were aligned to unigenes in the poly (A)^+^ RNA library using the sequence alignment program (UEA sRNA toolkit) with only perfect matches. Aligned sequences were used as anchors to extend 200 nt upstream or downstream of unigenes to predict their miRNA precursors. Precursor prediction was achieved using the miRCat program (UEA sRNA toolkit) and the secondary structure was analyzed by the mfold program (http://mfold.rna.albany.edu/?q=mfold/RNA-Folding-Form; Zuker, 2003). A sequence was considered as a valid miRNA candidate if its secondary structure met the following criteria according to Meyers et al. (2008): (1) a minimum free energy of the hairpin of –35 kcal/mol; (2) a maximum of four mismatches in miRNA and miRNA^*^ base-pairing; (3) no more than one asymmetrical bulge in the stem region with a size of 2 nt or less; (4) a ratio of miRNA and miRNA^*^ of 5:1 or higher; and (5) miRNA and miRNA^*^ duplexes showing 2 nt overhangs at the 3′ end. The relative frequencies of each miRNA family in the control and salt stress library were indicated by read per million (RPM). RPM was calculated as follows: the sum of reads that matched to each mature miRNA sequence of a miRNA precursor was divided by the total reads of 18–25 nt in the same library.

### Target predictions and annotations

psRNATarget (http://plantgrn.noble.org/psRNATarget/; Dai and Zhao, 2011) was applied to predict the putative targets of conserved and novel mcr-miRNAs using the ice plant transcriptome as described by Yim et al. (in preparation). The parameters for the target prediction were default settings and the maximum expectation was set to 3.0. Putative targets were annotated by BLASTX analysis against the nr protein database using default settings.

### Quantitative RT-PCR analysis of miRNAs and potential target genes

Poly(A)-tailing RT-PCR was performed according to the condition set by Shi and Chiang (2005). The poly(A)-tailing RT (PART) primers were designed accordingly and listed in Additional file 8. The PART primer consisted of an oligo(dT) sequence and introduced NV (N = A, T, C G; V = A, G, C) nucleotides at the 3′ end. The universal reverse primer ACATTATAGCGCGTAGTTAGA was located at the 5′ end of PART primer. U6 was used as an internal control and expressed equally well in control and salt-treated ice plant seedlings. Two micrograms total RNA isolated from control or salt-treated seedlings were polyadenylated by Poly(A)-tailing kit (Ambion, ThermoFisher Scientific, Waltham, MA). After polyadenylation, the products were reversed transcribed by adding 1 mM dNTP, 10 nM PART primer, U6-R primer, and 50U MultiScribe reverse transcriptase (Applied Biosystems, Life Technologies, Waltham, MA). The RT conditions were 16°C for 30 min, 42°C for 30 min, and 85°C for 5 min. Stem-loop RT-PCR was used to detect specific members of conserved miRNA families and novel miRNAs according to Varkonyi-Gasic et al. (2007). One microgram of total RNA from control or salt-treated seedlings was reverse transcribed using stem-loop RT primer by ImProm-II™ reverse transcriptase (Promega Corporation, Madison, WI). The RT was performed under the following conditions: 16°C for 10 min, 42°C for 10 min, and 70°C for 15 min. The same qPCR condition was used for Poly(A)-tailing and stem-loop RT products. Each qPCR reaction was performed in a final volume of 20 μL containing cDNA, 2X SYBR Premix Ex Taq (Takara Bio, Inc. Shiga, Japan), and 0.5 μM miRNA-specific forward primer and universal reverse primer using the following conditions: 95°C for 5 min, followed by 40 to 45 cycles of 95°C for 30 s, 55°C for 30 s, and 72°C for 30 s. U6 was used as an internal control. Product specificity was confirmed by a melting curve analysis (60–99°C) and gel electrophoresis after completion of thermal cycles. The qPCR reaction was performed in a Rotor-Gene Q (Qiagen, Redwood City, CA). The expression of each mcr-miRNA was repeated at least ten times and expressed as relative expression level (2^−ΔΔCT^) or fold change (–ΔΔCt = ΔCt control–ΔCt salt; Livak and Schmittgen, 2001). The method used for quantitation of target gene expression was similar to that used for miRNA. Total RNA was reverse transcribed using a random hexamer primer by ImProm-II™ reverse transcriptase (Promega) and cDNA was amplified by specific primer pairs designed for either conserved regions of the gene family or for target sequences recognized by specific miRNA (Additional file 8). qPCR was performed under the following conditions: 40–45 cycles at 95°C for 30 s, 55°C for 30 s, and 72°C for 30 s. Expression of *FNR1* (ferredoxin NADP^+^ reductase; GenBank: M25528.1) was used as an internal control for target gene expression. The relative transcript abundance of *FNR1* does not change in response to salt treatment (Cushman et al., 1989) and is used widely as an internal control for quantitation of salt-induced mRNA expression of ice plant.

### Degradome sequencing analysis

Degradome sequencing was performed according to the Parallel Analysis of the RNA Ends protocol (German et al., 2009). Twenty microgram of total control and salt-treated RNA samples were used for degradome sequencing. Library construction and degradome sequencing was performed by OneArray Bioscience (Taipei, Taiwan; Addo-Quaye et al., 2009). Sequencing data was analyzed by CleaveLand3.0 software and mapped to the ice plant transcriptome. A target plot (t-plot) was created to identify the true miRNA cleavage site from background noise.

## Results

### Sodium distribution in ice plant seedlings

**Figure 1:**
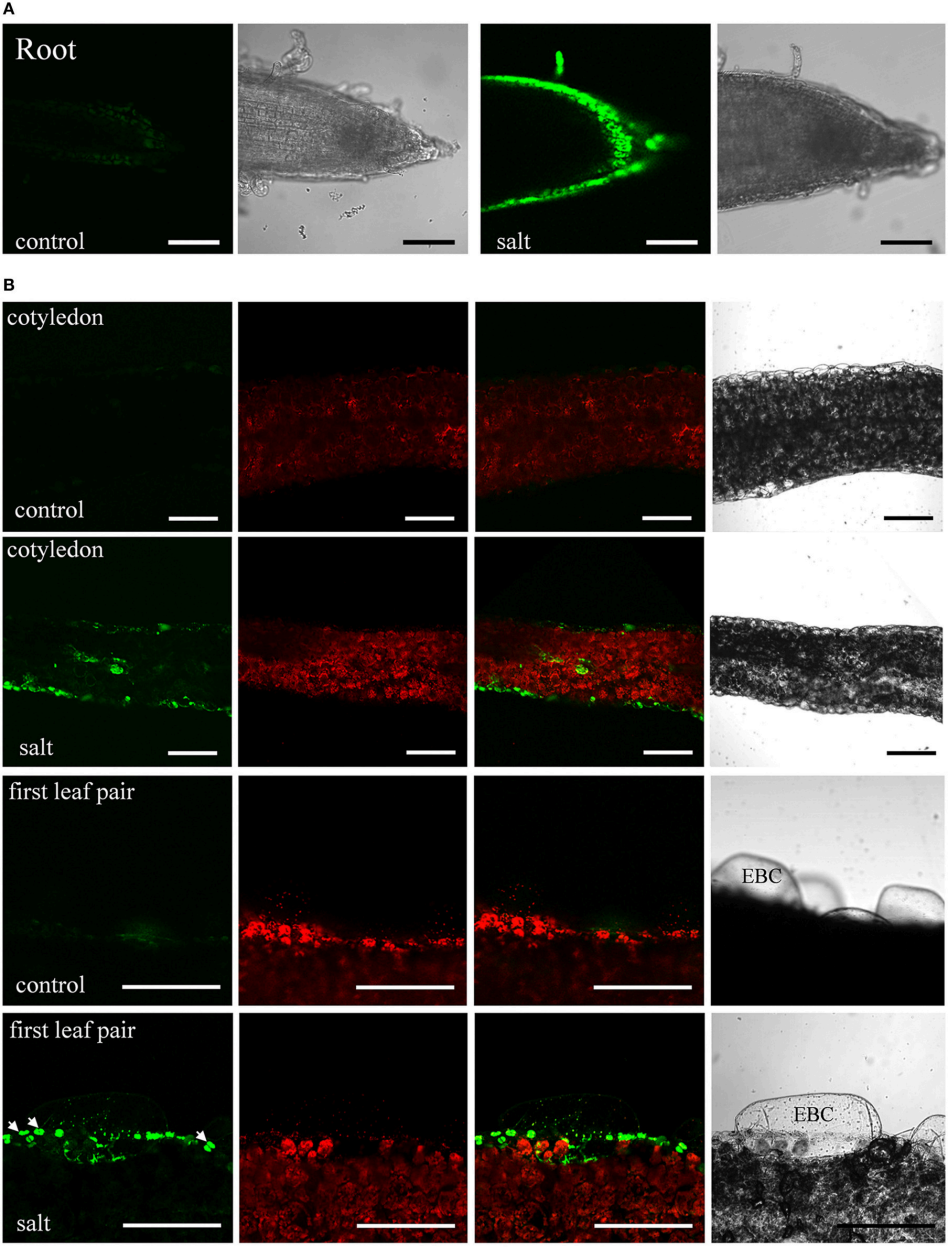
**Sodium distribution in 200 mM NaCl-treated ice plant seedlings**. Fluorescence detection of intact root and leaf slices stained with 10 μM Sodium Green for 30 min. **(A)** Roots of 1-week-old seedlings were treated with MS medium (control) or MS medium plus 200 mM NaCl (salt) for 6 h. Images of Sodium Green fluorescence are showed on the left and bright field images are showed on the right. **(B)** Fresh slices of 1-week-old cotyledons (first and second panel) and 2-week-old leaves (third and fourth panel) were treated with MS medium (control) or MS medium plus 200 mM NaCl (salt) for 24 h and were stained with Sodium Green for 30 min. Column 1 shows signals from Sodium Green; column 2 shows chlorophyll autofluorescence; column 3 shows merged images of column 1 and 2; column 4 shows bright field images. Arrows indicate the position of stomatal guard cell pairs. EBC, epidermal bladder cells. Bar = 1 mm.

The moderate salt tolerance of ice plant seedlings is determined genotypically. The initial responses of ice plant seedlings to sudden increases in salt concentration were examined. The distribution of Na^+^ in salt-treated tissues was detected by staining with Sodium Green, a fluorescent indicator of Na^+^ (Figure 1). Control seedlings germinated in a sodium-free MS medium accumulated Na^+^ in cells located in the root tips and the elongation zone (Figure 1A). The source of Na^+^ likely arose from salts sequestered in the dried seeds as the seeds were collected from plants that were salt-treated. The salt content was estimated at 5.52 mg Na^+^ per g dry weight, and is likely used as the primary osmoticum for cell expansion. In salt-treated seedlings, the distribution of Na^+^ changed after roots were immersed in 200 mM NaCl for 6 h (Figure 1A). High concentrations of Na^+^ were detected in the epidermal cells of the elongation zone, indicating that Na^+^ had been actively secreted; a similar pattern continued for 24 h (Additional file 1). Excess Na^+^ was also distributed to the aerial parts of seedlings. After 24 h of salt treatment, Na^+^ accumulation was observed in the epidermal cells and vascular bundles of the cotyledons and EBCs of the primary leaves (Figure 1B). We were very careful to allow only the roots and the lower regions of hypocotyls of the seedlings to be direct contact with the NaCl solution. Thus, the observed Sodium Green fluorescence must have arisen from Na^+^ transport. EBCs are a major salt-tolerant determinant for water and ion storage at adult and flowering stages. They remain appressed, but are metabolically active at the juvenile stage (Jou et al., 2007). A weak fluorescence was detected in EBCs of salt-treated primary leaves, while no signal was detected in control leaves. A prolonged salt treatment is likely needed to result in an increase in the amount of Na^+^ in these huge cells sufficient for detection. Strong fluorescent signals were also detected in guard cells (arrows in Figure 1B). Although high K^+^ accumulated in guard cells might change the dye-binding affinity of Na^+^, the results suggested that Na^+^ can possibly supplement K^+^ as an osmoticum to regulate stomatal opening in this halophyte. Overall, ice plant seedlings responded to an increased flux of Na^+^ by either secreting them into cells at the root surfaces or storing them in specialized leaf epidermal cells, similar to the responses found in adult plants.

### Features of the small RNA (sRNA) population of ice plant

**Figure 2:**
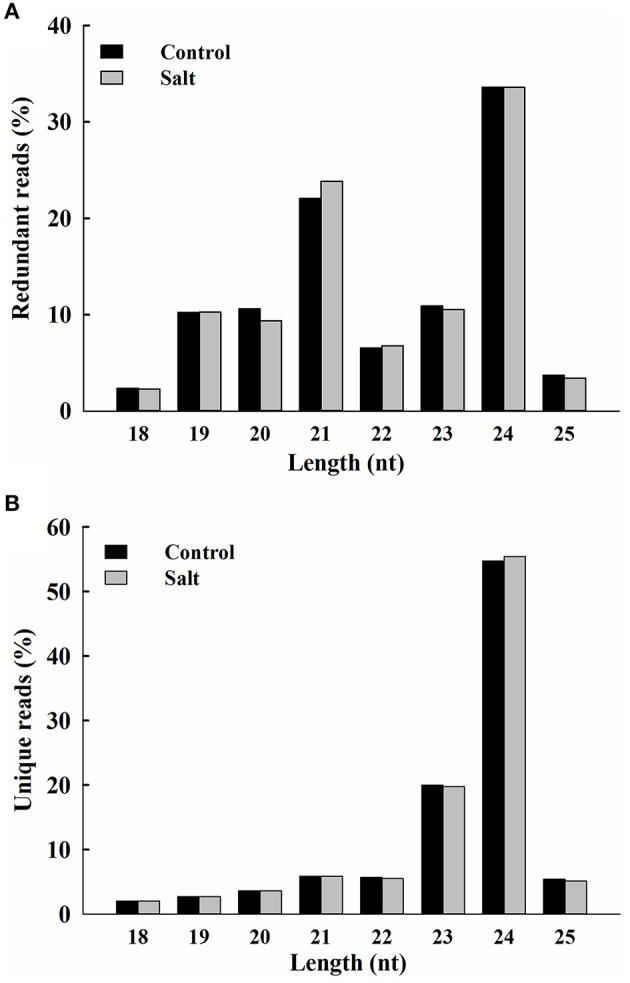
**Size distribution of ice plant small RNAs**. Redundant **(A)** and unique **(B)** sequences identified from control (black bar) and salt-treated (gray bar) seedlings and expressed as percentage of 18 to 25 nt total reads.

The rapid changes in Na^+^ distribution within epidermal cells in salt-stressed seedling roots suggest that ice plant responds in a timely fashion to sudden increases in Na^+^. Previously, deep-sequencing techniques have been used to study the transcriptome of ice plant seedlings, and transcriptional regulation in response to salt stress was observed (Tsukagoshi et al., 2015). In this study, RNA-Seq was used to analyze the changes in small RNA, focusing on the possible effects on miRNA-mediated post-transcriptional regulation. Small RNA populations from 3-day-old ice plant seedlings (control) and from the same-age seedlings treated for 6 h with 200 mM NaCl (salt) were sequenced. A total of 5,215,338 and 5,080,508 raw reads were generated from control and salt samples, respectively. After adaptor trimming, sequences not between 18 and 25 nt in length and sequences with ambiguous nucleotides or t/rRNAs were removed resulting in 3,089,326 and 3,157,610 high-quality redundant clean reads, from control and salt-treated seedlings, respectively, representing 1,106,388 and 1,118,741 unique sequences from control and salt-treated seedlings, respectively (Additional file 2). In the salt-treated seedlings, short-term salt treatment did not cause major alternations in the size distribution pattern of redundant or unique sRNAs (Figure 2), with the exception of a slightly lower proportion of 20-nt redundant reads and a slightly higher proportion of 21-nt redundant reads (Figure 2A). The most abundant small RNAs in both libraries were 24-nt long, which accounted for about one third of all sRNA species characterized, followed by those 21 nt in length, which about one fourth of all sRNAs characterized (Additional file 2). The highest sequence diversity was observed in the 24-nt sRNAs of unique reads, which accounted for about one half of unique sRNA species, followed by those with 23 nt, which accounted for about one fifth of unique sRNAs characterized (Additional file 2). Groups that were 21 and 22 nt in length had the highest percentage of small RNAs beginning with a U nucleotide, whereas the 24-nt group was enriched with sequences having an A at the 5′ end (Additional file 3). In addition to the analysis of small RNAs, the same sets of total RNA samples were subjected to poly (A)^+^ RNA transcriptome sequencing. The pooled mRNA-Seq yielded 53,031,502 reads, which were *de novo* assembled into 23,917 contigs with an average length of 1504 bp. The contigs ranged in size from 200 to 20,106 bp (N50 = 2125 bp), where 14,177 contigs were more than 1000 bp in length (Additional file 4). These contigs were used as a reference for following prediction of miRNA precursor.

### Identification of conserved and novel miRNAs in ice plant seedlings

To analyze the population of conserved miRNAs in ice plant seedlings, the 167,846 and 167,749 unique reads of 20–22 nt in length from control and salt libraries, respectively, were aligned with mature plant miRNA in the miRBase database using miRProf from the sRNA tool kit (Martínez et al., 2011). Only sequence reads that perfectly matched known mature plant miRNA or miRNA^*^ from miRBase were considered. The search identified 135 conserved miRNAs belonging to 21 miRNA families. All the miRNAs identified were highly conserved across diverse plant species. Detailed sequence polymorphism, read counts and potential targets are listed in Additional file 5. MiRNA156/157 was the largest family identified, with 27 members that were distinguished by internal nucleotide differences, followed by miR165/166 with 22 members, miR396 with 12 members, and miR159 with 11 members. The remaining 18 miRNA families contained between 2 and 10 members. The miR165/166 family had the highest number of reads in both control and salt-treated seedlings, followed by miR156/157 and miR159, which showed the second and third most abundant miRNA families, respectively (Additional file 5). All highly conserved miRNA families were detected in both control and salt samples. Certain families showed no significant change in relative read counts, whereas other families had higher or lower read counts in response to 6 h of salt treatment. Different members within the same miRNA family were also found to have opposite responses to salt treatment, indicating that ice plant seedlings express many conserved plant miRNAs that have diverse responses to salt stress.

**Figure 3:**
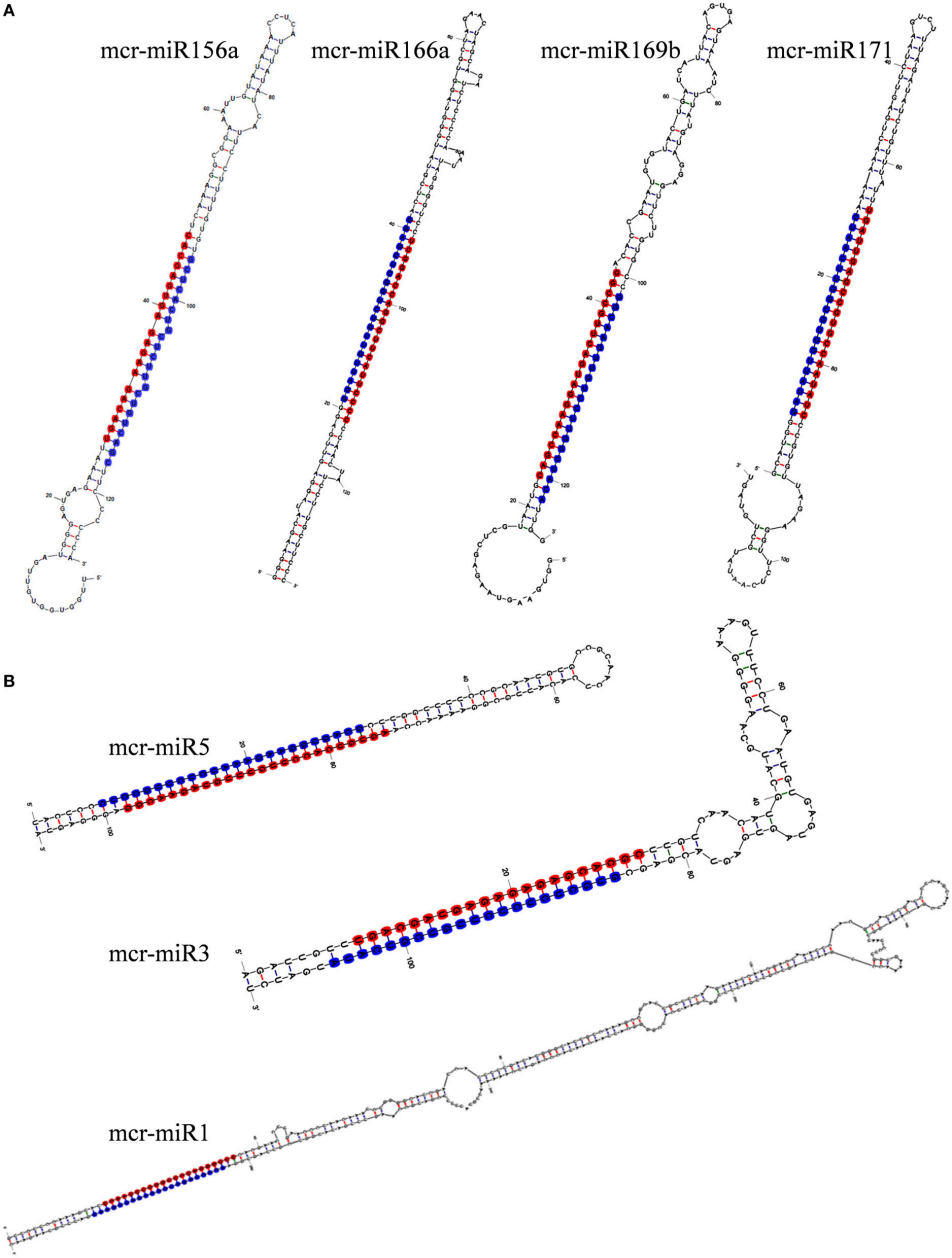
**Predicted hairpin structures of selected conserved (A) and novel (B) mcr-miRNA precursors**. Secondary structure prediction was carried out with the mfold program. The position of miRNA was labeled with red and miRNA^*^ with blue.

To identify *M. crystallinum* miRNAs (mcr-miRNAs), the 1,106,388 and 1,118,714 unique sequences from control and salt sRNA libraries were mapped to 23,917 contigs of the ice plant transcriptome using the sequence alignment program from the sRNA tool kit with a perfect match setting. Matched sequences were input to miRcat to predict precursor sequences with a hairpin structure. A sequence was considered as a valid mcr-miRNA precursor if its secondary structure met the ten criteria described in the Material and Methods. Thus, 19 conserved mcr-miRNAs belonging to 12 miRNA families (Table 1) were identified and some representative hairpin structures of miRNA precursors are shown in Figure 3. Most revealed a standard hairpin structure and the miRNA^*^ sequences were identified with two exceptions (mcr-miR160 and mcr-miR166b). All the miRNAs^*^ showed much lower reads than their corresponding miRNAs, consistent with the idea that miRNAs^*^ are degraded rapidly during the biogenesis of mature miRNAs. The minimum folding free energies (MFEs) of conserved mcr-miRNAs ranged from −43.9 to −103 kcal/mol and an average of −69.7 kcal/mol (Table 1), which was similar to the calculated average value for *Arabidopsis* miRNA precursors (−57 kcal/mol; Bonnet et al., 2004). The MFE index (MFEI), a criterion capable of distinguishing miRNAs from other types of coding or non-coding RNAs (Zhang et al., 2006), showed that these conserved mcr-miRNA precursors ranged from 0.82 to 1.50, which falls within the range of other known plant miRNA precursors. A list of conserved mcr-miRNA precursor sequences is provided in Supplementary Data Sheet 2. However, with more relaxed criteria, a greater number of miRNA would have been identified.

**Table 1:** **Conserved mcr-miRNA families identified in ice plant seedlings**.

**Family**	**Name**	**Sequence (5′ to 3′)**	**Strand (+/−)**	**Control RPM**	**Salt RPM**	**Hairpin Length**	**Length**	**MFEs**	**AMFEs**	**Hairpin G/C%**	**MFEI**	miRNA^*^ sequence
miR156/157	mcr-miR156a	TGACAGAAGAGAGTGAGCAC	−	7306.1	11515.7	20	126	−55.9	−43.33	44.96	0.96	GCTCACTGCTCTTGCTGTCAGC
	mcr-miR156b	TGACAGAAGAGAGTGAGCAC	−	6151.2	9904.6	20	110	−57.1	−57.10	52.00	1.10	GCTCACCCTCTCTCTGTCACC
	mcr-miR156c	CTGACAGAAGATAGAGAGCAC	+	613.1	543.4	21	128	−45.5	−56.88	45.00	1.26	GCTCTCTATCTTCTGTCATC
	mcr-miR156d	TTGACAGAAGATAGAGAGCAC	−	206.5	245.4	21	154	−86.61	−50.06	44.51	1.12	GCTCTCTATGCTTCTGTCATC
	mcr-miR156e	TGACAGAAGAGAGAGAGCAC	+	11	19.6	20	155	−67.4	−43.48	45.16	0.96	GCTCTCTCTTCTTCTGTCAAC
miR159	mcr-miR159a	TTTGGATTGAAGGGAGCTCC	−	3792.7	3910.2	20	209	−95.4	−45.65	40.67	1.12	AGCTCCCTTTGGTCCGAAAA
	mcr-miR159b	TTTGGATTGAAGGGAGCTCTA	+	1561.2	1307.6	21	194	−78.5	−44.10	42.70	1.03	GAGCTCCTTGAAGTCCAAAAG
miR160	mcr-miR160	TGCCTGGCTCCCTGTATGCCA	−	1.3	1	21	129	−52.2	−40.47	49.61	0.82	ND
miR162	mcr-miR162	TCGATAAACCTCTGCATCCAG	−	140.5	157.1	21	125	−43.9	−45.73	48.96	0.93	GGAGGCAGCGGTTCATCGATC
miR164	mcr-miR164	TGGAGAAGCAGGGCACGTGCA	−	2.3	1	21	258	−92	−35.66	39.53	0.90	CATGTGCCCCTCTTCACCATC
miR166	mcr-miR166a	TCGGACCAGGCTTCATTCCCC	+	56364.4	68848.6	21	132	−78	−59.09	54.55	1.08	GGATTGTTGTCTGGCTCGAGG
	mcr-miR166b	TCTCGGACCAGGCTTCATTCC	+	52852.6	58209.2	21	194	−79.1	−39.75	38.69	1.03	ND
miR168	mcr-miR168	TCGCTTGGTGCAGGTCGGGAA	−	104.2	101.3	21	164	−82.21	−50.13	56.10	0.89	CCCGCCTTGCATCAACTGAAT
miR169	mcr-miR169a	TAGCCAAGGATGACTTGCCT	−	116.5	151.4	20	143	−54.7	−38.25	42.66	0.90	GCAGTCATCATTGGCTAAG
	mcr-miR169b	CAGCCAAGGATGACTTGCCGG	+	1.6	1.9	21	126	−51.4	−40.79	46.83	0.87	GGCAAGTTGTCCTTGGCTACA
miR171	mcr-miR171	TGATTGAGCCGTGCCAATATC	−	49.8	39.9	21	116	−54	−59.34	39.56	1.50	GATATTGGTGCGGTTCAATC
miR319	mcr-miR319	TTGGACTGAAGGGAGCTCCCT	+	2410.9	2310	21	294	−103.1	−35.07	41.84	0.84	AGAGCTTTCTTCAGTCCACTC
miR396	mcr-miR396	TTCCACAGCTTTCTTGAACTG	+	439.9	509.6	21	215	−83	−38.60	31.63	1.22	GTTCAATAAAGCTGTGGGAAG
miR403	mcr-miR403	TTAGATTCACGCACAAACTCG	−	1321.3	1255.1	21	147	−64	−43.54	40.82	1.07	GATTTGTGCGTGAATCTAACG

RPM, reads per million; MFEs, minimal folding free energy (kcal/mol); AMFEs, adjusted MFEs (MFEs/ hairpin length)^*^100; MFEI, minimal folding free energy index; the modulus of adjusted MFEs divided by hairpin G/C %; ND, not detected.

**Figure 4:**
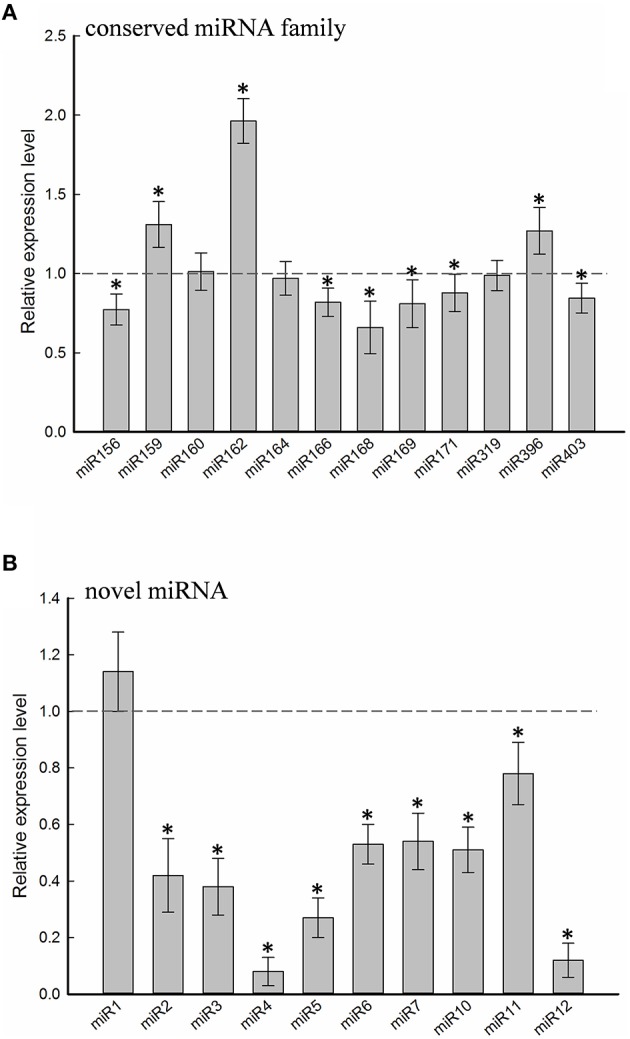
**Relative expression levels of conserved and novel mcr-miRNA**. Relative expression level of conserved miRNA families **(A)** and novel mcr-miRNAs **(B)** between control and salt-treated ice plant seedlings was detected by RT-qPCR and expressed as 2^−ΔΔCT^. Data was obtained from at least 5 independent experiments. Value higher than one indicates increased expression, while less than one indicates decreased expression under salt treatment. Significance of fold changes in the mcr-miRNA expression was determined using the Student's *t*-test. Asterisks represent significant difference between control and salt treatment at *P* ≤ 0.05.

Twelve of the high-confidence mcr-miRNA families were subjected to further analysis. First, poly(A)-tailing quantitative RT-PCR was used to examine the effect of salt on the expression of these 12 conserved mcr-miRNA families. This analysis confirmed that the expression of three mcr-miRNA families (e.g., miR160, miR164, and miR319) were unchanged in response to salt stress, whereas six showed reduced abundance (e.g., miR156, miR166, miR168, miR169, miR171, and miR403), and three showed greater abundance (e.g., miR159, miR162, and miR396) in response to salt treatment (Figure 4A). The greatest change in relative expression was less than two-fold indicating that short-term salt treatment did not have a dramatic impact on the expression of these conserved miRNA families.

In addition to identify conserved mcr-miRNAs, novel mcr-miRNAs precursors were predicted using the same approach. A total of 24 novel mcr-miRNAs with standard hairpin precursors and miRNA^*^ sequences were identified, which ranged from 21 to 24 nt in length (Table 2 and Figure 3). Eight novel miRNAs were 21 nt in length, six of which had uracil in the first position. The average MFEs and MFEI of these novel mcr-miRNA precursors was −122 kcal/mol and 1.34, respectively. The hairpin length of these novel mcr-miRNA precursors was more variable, ranging from 102 to 412 nt in length (Additional file 6). A list of novel mcr-miRNA precursor sequences is provided in Supplementary Data Sheet 2. These novel mcr-miRNAs are listed in Table 2 and were numbered according to their reads per million (RPM) from highest to lowest with the highest having only 141 RPM. Species-specific miRNAs are believed to have evolved more recently and are expressed at lower levels than well-conserved miRNAs (Allen et al., 2004). The relative expressions of 10 out of 24 predicted novel miRNAs were able to be detected by RT-qPCR in ice plant seedlings; all were found to display higher RPM according to NGS analysis. The relative abundances of the remaining 14 mcr-miRNAs were very low and could not be detected by RT-qPCR. The expression of nine novel miRNAs decreased under salt treatment (Figure 4B), suggesting that these potential species-specific miRNAs participate in the regulation of salt tolerance in ice plant.

**Table 2:** **Novel mcr-miRNA precursors identified in ice plant seedlings**.

**Name**	**Sequence (5′ to 3′)**	**Strand (+/−)**	**Control RPM**	**Salt RPM**	**Length**	**Hairpin Length**	**MFEs**	**AMFEs**	**Hairpin G/C%**	**MFEI**	miRNA^*^ sequence
mcr-miR1	TCAATTTGGGTTCTAGGGTTT	−	141.1	122.9	21	317	−135.5	−42.74	37.85	1.13	ACCCTAGATCCCAAATTGAAT
mcr-miR2	CCACCCGGGATCGTTTCGTGCAAC	−	70.2	67.1	24	286	−152.12	−53.19	36.36	1.46	AGTTGCATGAGACAGTCTTAGGTG
mcr-miR3	TGACGATGAGAGAGAGCACGC	−	65.4	50.4	21	111	−60.1	−54.14	45.95	1.18	GTGCTCTCTCTTGTCGTCATA
mcr-miR4	AATTAGGCCTAACGTCGGGTACCC	−	54.7	64	24	399	−210.6	−52.78	33.08	1.60	TTGGGTACCCGACGTTAGGTCTA
mcr miR5	AGTGTCACCTTGTTTGTAGAACGG	−	38.5	24.4	24	106	−82.7	−78.02	49.06	1.59	CTCCGTTCTCTAAAGAAGGTGACG
mcr-miR6	TTCGTGCTGATAACGTGTTGAA	−	32.7	26.6	22	233	−139.3	−59.79	28.33	2.11	CAACACTTTATCAGCACGAATC
mcr-miR7	CACCTTGTTTGTAGAACGGAGGGA	+	25.6	17.4	24	102	−50.9	−49.90	44.12	1.13	TACTCCCTCCGTTCTCTAAAGAA
mcr-miR8	TCTTTTTATAGAGGAATGCCTC	−	24	25.7	22	108	−45.11	−41.77	33.33	1.25	GGCCTTCCTCTTAAAAAGATG
mcr-miR9	TCGGAAAATGACTTAAGGGGT	+	16.2	10.1	21	300	−134.2	−55.92	36.25	1.54	CACCTTAAGTCGTTTTTCGCC
mcr-miR10	CCCGGACCATGTAATAATTGCTC	+	17.8	14.3	23	173	−98.1	−56.71	45.09	1.26	GAAATTATTGTATGGTCCCGGAC
mcr-miR11	TTCCGGCAGGTTGTCCTTGGC	+	18.1	15.8	21	194	−54.95	−28.32	36.60	0.77	CAGCCAAGGATGACTTGCCGG
mcr-miR12	ATAGGGCGAGATTGACAAACC	−	11	12	21	164	−78.7	−55.82	40.43	1.38	TTGGTTTGTCAATCTCGATCT
mcr-miR13	CCTAAAGGTTGGATTATTGGCATC	+	12	13	24	412	−234.19	−56.84	35.68	1.59	ATGCCAATAATCCAACCTTTGGGC
mcr-miR14	TTGGCCGGAAAATTGAACAAG	−	9.1	14.3	21	313	−176.99	−56.55	35.46	1.59	CCCTTGTTCAATTTTCTGACC
mcr-miR15	CTGAAGCGTTTGGGGGAACTC	−	7.1	4.4	21	104	−44.95	−43.22	48.08	0.90	GTTCCTCACAGCACTTCATTG
mcr-miR16	AAGGGCTCGCTTGGATTGGGGGGA	−	6.2	11.1	24	372	−240.3	−64.60	45.70	1.41	TTTCCCTCAATCCAAGCGGGGCCT
mcr-miR17	ATCACCGGTGGCGAATTTTTGGGC	−	4.2	4.8	24	360	−197.87	−54.96	35.28	1.56	GAGCCCAAAAATTCGCCGCCGGTG
mcr-miR18	ACACCAACGAGGACTTTGAAACAC	+	3.2	4.4	24	176	−131.1	−74.49	47.16	1.58	GTTTCAAAGTCTTCGTTGGTGTCC
mcr-miR19	CTAAAGCGACAGTTATTTTGAGAC	−	2.9	2.2	24	168	−53.17	−31.65	33.33	0.95	CCGTCTCAAAATAACTGTCGCTTT
mcr-miR20	ATCACCGGTGGCGAATTTTTGGAC	−	2.9	1.9	24	360	−199.6	−55.44	36.94	1.50	GAGTCCAAAAATTCGCCGTCGGTG
mcr-miR21	TTTCGTGCAACTATAGTGAAAGGT	−	2.3	1	24	261	−122.72	−47.02	35.63	1.32	TTTCACTATAGTTGCATGAGACAG
mcr-miR22	TAAAGTTGGGATGTTCCTACC	−	1.9	2.2	21	309	−173.3	−56.08	47.25	1.19	TAGGAACATCCCGACTTTACT
mcr-miR23	GGACAAATAATTTGGGACGGAGGG	+	1.6	0.3	24	127	−67.1	−52.83	39.37	1.34	CTCCGTGTCCAATTATTTGTCTGC
mcr-miR24	TGTGAGAGAAGGGACCATAGGTTC	+	0.6	4.1	24	145	−44.8	−30.90	34.48	0.90	TCGGACCAATGCTCCCTCTTTCAT

RPM, reads per million; MFEs, minimal folding free energy (kcal/mol); AMFEs, adjusted MFEs (MFEs/hairpin length)^*^100; MFEI, minimal folding free energy index; the modulus of adjusted MFEs divided by hairpin G/C %.

### Prediction of mcr-miRNA targets

To understand the function of conserved and novel mcr-miRNAs, putative target genes were predicted using psRNATarget with default parameters. Details of the ice plant transcriptome used in searching mcr-miRNA are described by Yim et al. (in preparation). Potential targets of conserved and novel mcr-miRNAs were predicted with a 0–3 expectation-score cutoff threshold and are listed in Table 3. Potential target genes appeared to be involved in a broad range of biological processes. The targets for most conserved mcr-miRNAs were genes encoding transcription factors, such as miR156 target squamosa promoter-binding protein-like (SPL); miR159 and miR319, both target MYB transcription factors; miR160, which targets auxin response factor (ARF); and miR164, which targets NAM (no apical meristem); ATAF (Arabidopsis transcription activation factor); CUC (cup-shaped cotyledon) transcription factor (NAC); miR166, which targets the homeobox-leucine zipper family protein (HD-ZIP); and miR169, which targets nuclear factor Y subunit A (NF-YA). These examples support the idea that conserved plant miRNAs are involved in the regulation of a large gene expression network. Degradome sequencing analysis identified that mcr-miR164, 166b, 169a, b, and 403 specifically cleaved the transcripts of their predicted target genes (Additional file 7), proving miRNA-mediated target cleavage in ice plant seedlings. In addition to the genes coding for transcription factors, non-conserved targets were also predicted to be regulated by conserved mcr-miRNAs. Although the relevance of miRNA-target interaction needs to be confirmed, several predicted targets were shown to function in osmotic stress, for example, glutathione-regulated potassium-efflux protein (mcr-miR160), early responsive to dehydration stress protein ERD4 (mcr-miR171), and heat shock protein 80 (mcr-miR396).

**Table 3:** **Predicted targets of conserved and novel mcr-miRNA**.

**miRNA**	**Predicted function**	**Expectation**	**Location**	**Inhibition**	**Target gene**
mcr-miR156	Squamosa promoter-binding protein-like (SPL)	0	ORF	Cleavage	tr_25478; 92930; 247074; 130241; 134702; 242974
	Glutaredoxin family protein	2.5	ORF	Cleavage	tr_222987
	NAD(P)-binding Rossmann-fold superfamily protein	3	ORF	Cleavage	tr_112639; 112626
	Oxophytodienoate-reductase 3	3	ORF	Cleavage	tr_8553
	Tetratricopeptide repeat-like superfamily protein	3	ORF	Cleavage	tr_211611
	NAD(P)-dehydrogenase	3	ORF	Cleavage	tr_20823
	F-box family protein	3	ORF	Translation	tr_250578
	Gibberellin receptor GID1B	3	5′ UTR	Translation	tr_131891
mcr-miR159	MYB domain protein 33	2.5	ORF	Cleavage	tr_247776
	Cytidinediphosphate diacylglycerol synthase 2	2.5	5′ UTR	Cleavage	tr_24710
	Polyketide cyclase/dehydrase lipid transport protein	3	ORF	Cleavage	tr_247457
	Histone H2A protein 9	3	ORF	Cleavage	tr_31155
	ATP binding cassette subfamily B1	3	ORF	Cleavage	tr_75146
	RNA ligase	3	ORF	Cleavage	tr_251381
	Glycosylhydrolase superfamily protein	3	ORF	Cleavage	tr_63329
mcr-miR160	Auxin response factor (ARF) 16	0.5	ORF	Cleavage	tr_131045
	Glutathione-regulated potassium-efflux protein	2.5	ORF	Cleavage	tr_29190
mcr-miR162	Dicer like-1	2	ORF	Cleavage	tr_87681
	Pyridoxamine 5′-phosphate oxidase family protein	3	ORF	Translation	tr_128506
mcr-miR164	NAC domain containing protein	1	ORF	Cleavage	tr_20281
mcr-miR166	Homeobox-leucine zipper family protein (HD-ZIP)	1.5	ORF	Cleavage	tr_23906; 16028
	NB-ARC domain-containing disease resistance protein	3	ORF	Translation	tr_6344
mcr-miR168	Argonuate 1	3.5	ORF	Cleavage	tr_23616
	Cytochrome P450, family 76	3	ORF	Translation	tr_34562; 106564
mcr-miR169	Nuclear factor Y subunit A (NF-YA)	2	3′ UTR	Cleavage	tr_241835
	Haloacid dehydrogenase	3	ORF	Cleavage	tr_78265
mcr-miR171	Scarecrow-like protein (SCL) 6	0.5	ORF	Cleavage	tr_129576
	Early-responsive to dehydration stress protein (ERD4)	3	ORF	Cleavage	tr_88429
	Cysteine-rich RLK (Receptor-like protein kinase) 25	3	ORF	Cleavage	tr_72609
	Subtilisin-like serine protease 3	3	ORF	Cleavage	tr_23970
	ARM repeat superfamily protein	3	ORF	Cleavage	tr_25709
mcr-miR319	MYB domain protein 33	2.5	ORF	Cleavage	tr_247776
	TCP-4 transcription factor^*^	4	ORF	Cleavage	tr_248183
mcr-miR396	Growth-regulating factor (GRF) 2	2	ORF	Cleavage	tr_20229
	Alfin-like 3	2.5	ORF	Cleavage	tr_86713
	Heat shock protein 81.4	3	ORF	Translation	tr_24595
	Ribosomal protein L1p/L10e family	3	ORF	Cleavage	tr_27243
	Zinc finger protein-related	3	ORF	Cleavage	tr_8491
	F-box protein	3	3′ UTR	Cleavage	tr_24495
	Proteasome family protein	3	3′ UTR	Translation	tr_25619
mcr-miR403	Argonaute 2	1	3′ UTR	Cleavage	tr_81134
	Ubiquitin-specific protease	3	3′ UTR	Translation	tr_23630
	ADP-ribosylation factor GTPase-activating protein	3	ORF	Translation	tr_25728
mcr-miR1	Mitochondrial transcription termination factor protein	1.5	ORF	Cleavage	tr_26362; 27606
	Germin-like protein	3	ORF	Cleavage	tr_140178
	Thioredoxin superfamily protein	3	ORF	Cleavage	tr_126594
	Ubiquitin-specific protease	3	ORF	Cleavage	tr_90873
	BED zinc finger; hAT family dimerisation domain	3	ORF	Translation	tr_16919
mcr-miR3	Sodium/myo-inositol symporter (ITR) 3	3	ORF	Cleavage	tr_16378
	Cytochrome P450, family 81	3	ORF	Translation	tr_127844
mcr-miR4	Leucine-rich repeat transmembrane protein kinase	0.5	3′ UTR	Cleavage	tr_124137
	Multi-antimicrobial extrusion (MATE) efflux protein	0.5	3′ UTR	Cleavage	tr_15134
	Protein translocase subunit SecA, chloroplastic	1.5	3′ UTR	Cleavage	tr_77781
	Protein kinase superfamily protein	1.5	3′ UTR	Cleavage	tr_91240; 91241; 91239
	NAD(P)-binding Rossmann-fold superfamily protein	3	ORF	Cleavage	tr_28492
mcr-miR5	Phosphofructokinase 3 (PFK)	2	ORF	Cleavage	tr_24929
	Tetratricopeptide repeat-like superfamily protein	2	ORF	Cleavage	tr_251318
mcr-miR10	Germin 3	3	3′ UTR	Cleavage	tr_32811
mcr-miR11	Ribosomal protein L34	2.5	ORF	Cleavage	tr_32249
mcr-miR12	Arginyl-tRNA synthetase	3	ORF	Translation	tr_249031
	Translin family protein	3	ORF	Cleavage	tr_131509

* *miR319/TCP-4 pair was aligned manually*.

**Figure 5:**
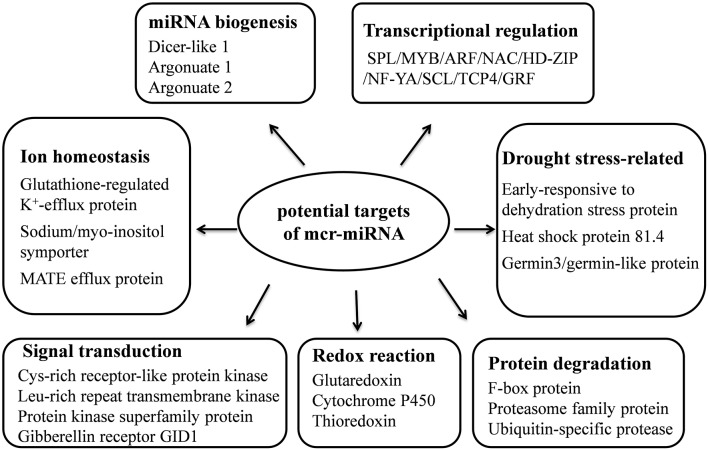
**Summary of functions of potential target genes identified in ice plant seedlings**.

Unlike conserved mcr-miRNAs, the potential targets of novel mcr-miRNAs were not enriched in transcription factors. Many of them are metabolic enzymes and stress-related proteins (Table 3). Among them, genes coding for sodium/myo-inositol symporter (ITR) is a potential mcr-miR3 target and germin/germin-like proteins are potential mcr-miR1 and miR10 targets with both genes known to be involved in the salt tolerant mechanisms of ice plant (Michalowski and Bohnert, 1992; Nelson et al., 1999; Chauhan et al., 2000). The known functions of potential targets of the conserved and novel miRNAs were categorized into seven biological functional groups (Figure 5). In addition to the well-characterized transcriptional regulation and miRNA biogenesis, targets known to play roles in ion homeostasis, drought tolerance, signal transduction, redox reaction, and protein degradation were identified (Figure 5). The current analysis of mcr-miRNAs in ice plant seedlings revealed a diverse set of target genes with putative functional roles in the response to short-term salt stress treatment.

### Salt-induced changes in the expression of mcr-miRNAs and potential target genes

**Figure 6:**
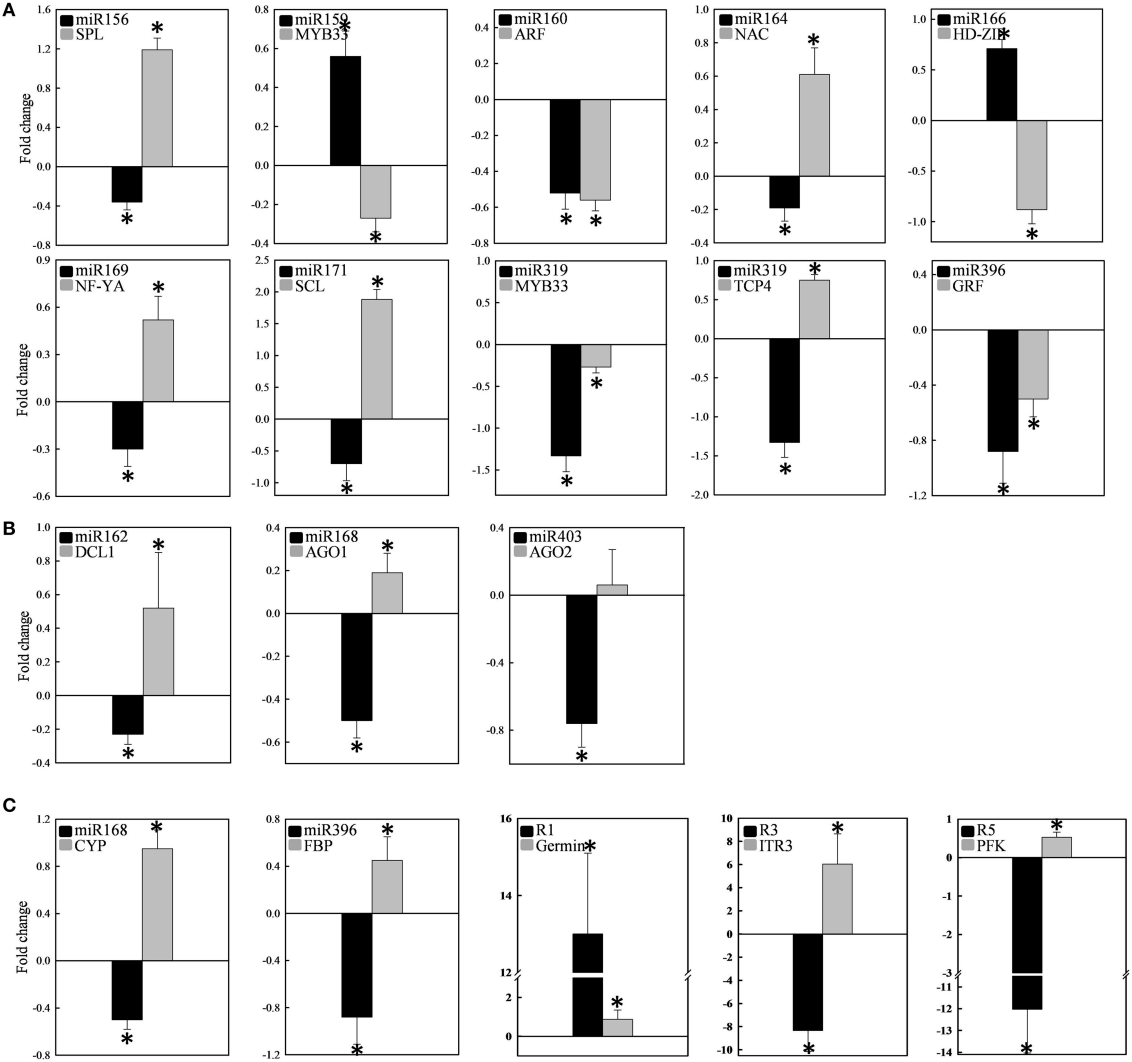
**Expression of 12 conserved and 3 novel mcr-miRNAs and their potential target genes**. The expression fold change was determined by RT-qPCR and expressed as -ΔΔCt. The expression of individual miRNA (black bar) and its target (gray bar) categorized into transcription factors **(A)**, miRNA biogenesis **(B)**, and stress-related **(C)** was shown in the same plot. Measurement was performed from at least 10 independent experiments. Significance of fold changes in the miRNA and target gene expression was determined using the Student's *t*-test. Asterisks represent significant difference between control and salt treatment at *P* ≤ 0.05. Values higher than zero indicate increased expression, whereas values less than zero indicate decreased expression under salt treatment.

**Figure 7:**
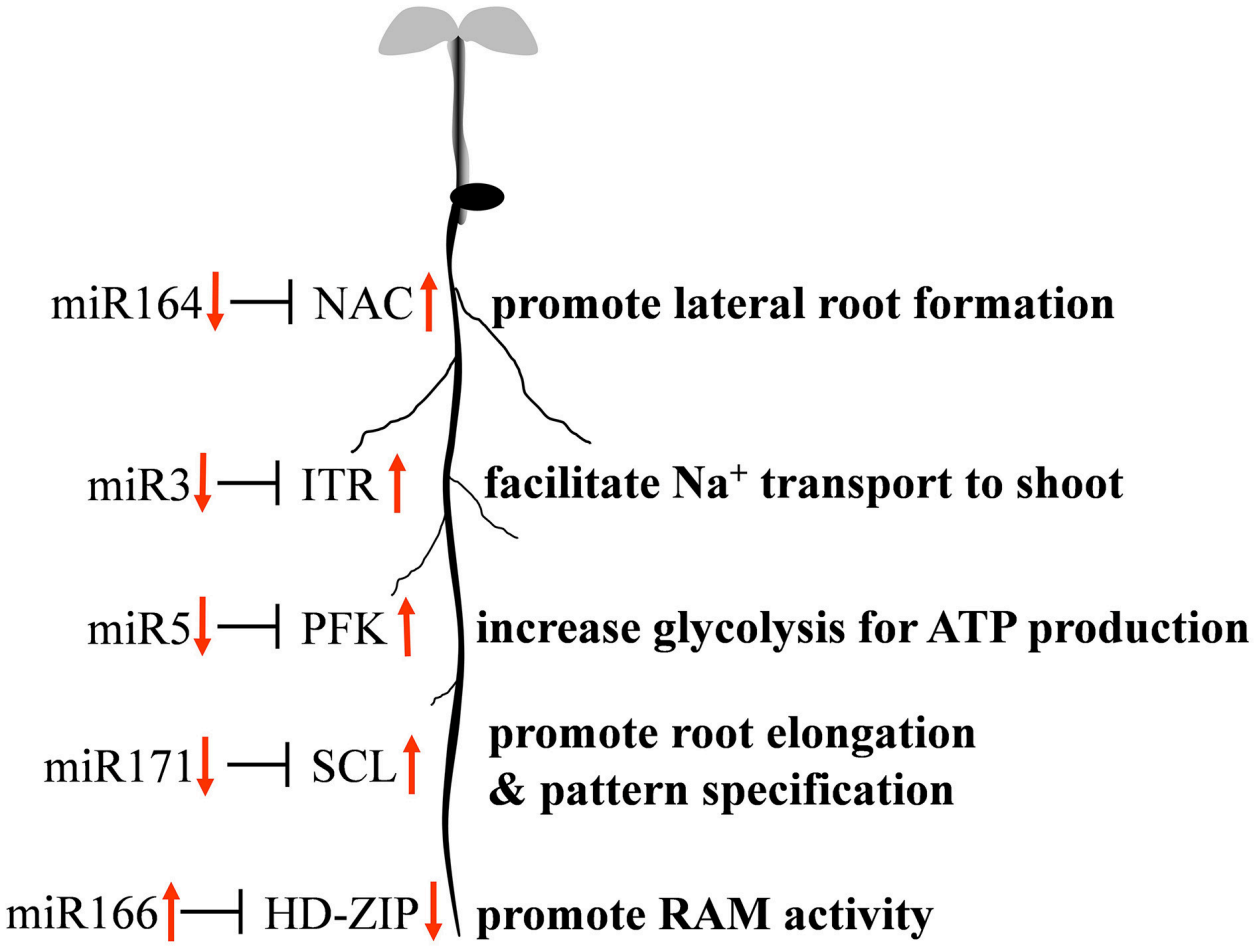
**Proposed model of mcr-miRNA-mediated initial responses to salinity in ice plant roots**.

Quantitative RT-PCR was used to examine the expression profile of selected mcr-miRNAs and their potential targets. Primers designed for stem-loop RT-PCR were used to detect the expression of a specific member of each miRNA family. Each primer pair amplified a single band using conventional RT-PCR demonstrating the specificity of the stem-loop primer. PCR products were subjected to DNA sequencing and matched perfectly to the corresponding miRNA sequence (data not shown). The expression level of miRNAs and target genes in the control and salt treatment was quantified and their relative expression was presented as fold changes (Figure 6). The expression of mcr-miR159b and 166b were increased, whereas the expression of rest of the mcr-miRNAs was decreased by salt treatment. For miRNA-transcription factor interactions, seven out of ten combinations showed reciprocally correlated expression patterns (Figure 6A). Mcr-miR319 was predicted to target two genes coding for the MYB domain protein 33 (MYB33) and TCP4. Whereas the expression of mcr-miR319 was decreased by salt treatment, *TCP4* showed increased expression. The expression of *MYB33* remained nearly unchanged by salt treatment, suggesting that miR319 selectively regulates *TCP4* expression in salt-stressed seedlings. The expression of three mcr-miRNAs, miR162, 168, and 403, which are likely involved in the regulation of miRNA biogenesis, all decreased following salt treatment, whereas the expression of their corresponding targets (i.e., *DCL1, AGO1*, and *AGO2*) were all increased (Figure 6B). The expression of 4 out of 5 non-conserved targets, selected from different functional groups, including three novel mcr-miRNA targets, were increased by salt treatment (Figure 6C). After examining the expressions of mcr-miRNAs and corresponding targets, traits related to promotion of root growth and maintenance of Na^+^ homeostasis are summarized in Figure 7.

## Discussion

The halophytic ice plant has been used as a model for salt tolerance for more than 40 years. Early studies on biochemical characterization and gene expression of the CAM pathway (Winter et al., 1982; Cushman et al., 1989) and sodium compartmentation (Ratajczak et al., 1994; Barkla et al., 1995) have provided a solid foundation for transcriptomic (Kore-eda et al., 2004; Cushman et al., 2008), proteomic (Jou et al., 2007; Barkla et al., 2009, 2012; Cosentino et al., 2013), and metabolomic and ionomic (Barkla and Vera-Estrella, 2015; Barkla et al., 2016) profiling of ice plant. Using RNA-Seq techniques, several studies deciphering ice plant transcriptome have revealed salt-induced changes in gene expression at different developmental stages (Tsukagoshi et al., 2015; Yim et al., in preparation) and in specific salt storage cells (Oh et al., 2015). Expression of several genes categorized as “response to heat stress” and “response to salt stress” were increased in ice plant seedlings treated with 140 mM NaCl for 24 h (Tsukagoshi et al., 2015). Previously, root tips of 3-day-old ice plant seedlings were used as a model to examine the salt-induced change of cellular protein localization (Chiang et al., 2013). We used these same treatment conditions to monitor intracellular levels of Na^+^ and found that ice plant roots likely secreted Na^+^ and had an efficient transport system to relocate Na^+^ to the aerial part of the plant (Figure 1). The expression of *MItr1*, a gene coding for H^+^-Na^+^/myo-inositol symporter, was highly induced in seedlings treated with salt for 12 h (Chauhan et al., 2000). Ice plant roots likely possess an innate Na^+^/K^+^ homeostatic mechanism such that high external Na^+^ causes this system to become highly expressed. However, future experiments are needed to resolve the exact hierarchical nature of this regulation whether it be via transcriptional, post-transcriptional, translational, or post-translational regulatory mechanisms.

The distribution of ice plant seedling sRNA was similar to the majority of plant species reported so far, where 24-nt sRNA was the most abundant miRNA species identified. In addition to model plants, such as *Arabidopsis* (Lu et al., 2005), *Oryza sativa* (Morin et al., 2008), and *Zea may* (Wang et al., 2011), 24-nt sRNA comprise 50–60% of known sRNA types, as well as make up 55 and 35% of the total sRNA in the halophytic plants chenopod *S. europaea* (Feng et al., 2015) and mangrove *A. marina* (Khraiwesh et al., 2013), respectively. These 24-nt sRNAs are known to be involved in heterochromatin modification directed by AGO4 (Havecker et al., 2010). AGO4 was identified previously as nuclear-localized in ice plant and was thought to be regulated through ubiquitination (Li et al., 2014). Salt treatment in ice plant did not cause significant changes in the abundance of 24-nt sRNA (Figure 2); however, the relevance of AGO4-catalyzed DNA methylation needs to be further clarified. The second most abundant class in ice plant seedlings was 21-nt sRNA, which is primarily composed by miRNA and siRNA (Mi et al., 2008). The most significant increase in sRNA abundance was associated with 21-nt species after 6 h of salt treatment (Figure 2). The genes coding for miRNA biogenesis, *DCL1, AGO1*, and *AGO2*, were found to show increased abundance (Figure 6), suggesting that the salt-induced miRNA population might contribute to the increase in 21-nt sRNA in ice plant seedlings under salt treatment.

By searching the sRNA library, more than 100 highly conserved miRNAs belonging to 21 miRNA families in dark-grown seedlings, a rapid growth stage, were discovered. Thus, the conserved miRNAs identified might play roles in root growth and development. A set of 12 conserved mcr-miRNAs were analyzed whose precursors can fold into standard hairpin structures. Seven mcr-miRNAs showed reciprocal expression patterns to their predicted transcription factor targets (Figure 6), many of whose functions have been identified in root development. In *Arabidopsis*, miR166/165 promotes root growth by targeting *HD-ZIP III* transcript, and overexpression of *MIR166* leads to an increased cell division in root apical meristem (RAM; Singh et al., 2014). Salt treatment resulted in an increased in the relative abundance of mcr-miR166, whereas the relative abundance of *HD-ZIP III* transcripts decreased, suggesting that RAM activity might increase in ice plant root tips. However, additional analysis will be needed to confirm this possibility. In the formation of lateral roots, the function of miR164 is to target direct cleavage of the *NAC* transcript. *Arabidopsis* mutants with a reduced amount of miR164 expressed more *NAC1* mRNA and produced more lateral roots (Guo et al., 2005). Salt treatment decreased mcr-miR164 and increased *NAC* expression suggesting that the formation of lateral roots in ice plant seedlings might be mediated by these miRNAs. The *SCL* family is targeted by miR171, which controls root growth and radial patterning, particularly for the specification of the endodermis (Helariutta et al., 2000). Overexpression of *MIR171* resulted in the severe inhibition of root elongation in *Arabidopsis* seedlings (Wang et al., 2010). Salt treatment decreased mcr-miR171 and increased expression of the *SCL* family in ice plant roots, suggesting that root elongation and root pattern formation might undergo modulation through the action of this miRNA; however, additional experimentation is needed to confirm this possibility. Tsukagoshi et al. (2015) showed that the roots of ice plant seedling continued to grow in a high rate when treated with 140 mM NaCl for 24 h, while the growth of Arabidopsis roots was completely inhibited. Several key regulatory events likely to be associated with root growth and salinity adaptation are summarized in Figure 7; however, these processes are likely to be more complex than the model indicates. For example, although mcr-miR160 and miR396 did not show opposite expression patterns to their potential targets under salt treatment, their roles in the regulation of cell division, cell differentiation (miR160), and cell elongation (miR396) have been clearly documented (Wang et al., 2005; Bao et al., 2014).

Non-conventional miRNAs are known to function in ion homeostasis, osmotic adjustment, and ROS elimination. Mcr-miR1 is predicted to target a gene coding for a germin-like protein that has been found to show increased expression in ice plant roots after 6 h of salt stress (Michalowski and Bohnert, 1992). Monocot germin-like proteins have oxalate oxidase activity that generates H_2_O_2_ by degrading oxalic acid (Caliskan, 2000). Because its activity simultaneously releases Ca^2+^ from calcium oxalate and generates H_2_O_2_, germin family proteins have been proposed to function in cell growth and development. The function of germin in salt stress was also proposed (Caliskan, 2011). In Figure 6, the expression of genes coding germin-like proteins was salt-induced, but mcr-miR1 did not show opposing expression patterns. Therefore, the post-transcriptional regulation of germin-like genes in salt-stressed ice plant roots requires further examination. Novel mcr-miR3 is predicted to target transcripts encoding the sodium/myo-inositol symporter gene *Itr*, which has been identified to function in removing Na^+^ from root to leaf vacuoles in ice plant. In this study, a rapid relocation of Na^+^ from the root to the leaf guard cells and bladder cells was inferred from Sodium Green patterns of detection. There are three *Itr* genes identified in ice plant: *Itr1* is highly expressed in roots 6 h after salt stress, *Itr2* is weakly expressed in salt-stressed leaves (Chauhan et al., 2000), and to date, there is no information on the expression of *Itr3*. Because the predicted target of mcr-miR3 is *Itr3* and a five-fold increase in *Itr3* expression was accompanied by reduced expression of mcr-miR3 (Figure 6), the putative miRNA-mediated regulation of *Itr* expression is an attractive subject for future investigations. Mcr-miR5 is predicted to target genes coding for phosphofructokinase 3 (*PFK3*), the committed step of glycolysis. *PFK3* is a potential target of mcr-miR5, and it is an ATP-dependent phosphofructokinase that is highly expressed in roots and is heat-inducible in *Arabidopsis* (Mustroph et al., 2007). The increased expression of *PFK3* transcripts observed here suggests that this energy-production pathway might play important roles in providing ATP for growth and maintaining ion homeostasis under sudden influxes of Na^+^. Plant miRNAs have been conserved over evolutionary timescales. Some sequence variations in a few nucleotide positions provide the opportunity for miRNAs to target non-conserved mRNAs, exhibiting species-specific regulatory patterns (Axtell and Bowman, 2008). Using NGS techniques to detect rare, small RNA species, a large number of novel miRNAs have been reported as having species-specific behavior; however, more studies are needed to demonstrate the mechanisms and functions of novel mcr-miRNAs and their targets.

In conclusion, when ice plants were germinated in a Na^+^-free medium, they used NaCl stored in their seeds for cell expansion. The characterization and determination of the expression patterns of a set of known miRNAs suggests that root responses to salinity are likely to be modulated, in part, by miRNA-mediated post-transcriptional regulatory events. The discovery of new, species-specific miRNA also suggests that miRNA-mediated post-transcriptional regulatory events participate in responses to sudden increases in salt. Cellular metabolism, osmotic adjustment, ion transport, and other processes were likely subjected to miRNA-mediated regulation. Characterization of mcr-miRNA and their target genes has thus added new insights into our understanding of the putative mechanisms involved in miRNA-regulated salt tolerance in this halophyte.

## Author contributions

CC performed ice plant miRNA search, characterization and expression, and drafted the manuscript. WY assembled ice plant transcriptome for target identification and searched for miRNA precursors. YS helped to analyze the small RNA sequencing results and designed the RT-qPCR experiments. MO and TM participated in the design of sodium staining in seedling roots and leaf sections. JC provided ice plant transcriptome and commented on the manuscript. HY conceived of the study, designed the experiments, analyzed the data and finalized the manuscript. All authors read and approved the final manuscript.

## Funding

This study was supported by the Ministry of Science and Technology, Taiwan, under the grant number MOST 103-2311-B-005 -003 to HY. JC acknowledges support from the National Science Foundation (Grant number IOS-084373), the University of Nevada Agricultural Experiment Station, and the Department of Energy, Office of Science, Genomic Science Program (Contract Number DE-SC0008834).

### Conflict of interest statement

The authors declare that the research was conducted in the absence of any commercial or financial relationships that could be construed as a potential conflict of interest.
