# Supplementary material for: Identification of Ice Plant (Mesembryanthemum crystallinum L.) MicroRNAs Using RNA-Seq and Their Putative Roles in High Salinity Responses in Seedlings
Source: Front Plant Sci. 2016 Aug 9;7:1143. doi: 10.3389/fpls.2016.01143 (PMC4977306; doi:10.3389/fpls.2016.01143)
Supplement: Supplementary file 1 [file DataSheet1.pdf]

Supplementary Material

Identification of ice plant (*Mesembryanthemum crystallinum* L.) microRNAs using RNA-Seq and their putative roles in high salinity responses in seedlings

Chih-Pin Chiang[1], Won Cheol Yim[2], Ying-Hsuan Sun[3], Miwa Ohnishi[4], Tetsuro Mimura[4], John C. Cushman[2]*, Hungchen E. Yen[1]*

*** Correspondence:**
HE Yen (responsible for content and experimental procedures in this article)
heyen@dragon.nchu.edu.tw
JC Cushman (responsible for transcriptome information requests)
jcushman@unr.edu

Additional file 1. Sodium distribution in 24 h 200 mM NaCl-treated ice plant roots.

Additional file 2. Summary of small RNA sequencing (A) and proportion distribution of 18-25 nt clean reads (B)

Additional file 3. Percentage of unique sequences starting with an A, C, G, and U in each size group in control (A) and salt (B) seedlings

Additional file 4. Summary of poly $(A)^+$ RNA sequencing in ice plant seedlings Additional file 5. Sequences and length polymorphisms of conserved miRNAs in ice plant seedlings. Boldface indicates the sequences listed in Table 1.

Additional file 6. Sequences and predicted hairpin structures of conserved and novel mcr-miRNAs.

Additional file 7. Target plots (t-plots) show mcr-miR164 (A), 166b (B), 169a, b (C), and 403 (D) targeting to corresponding genes using degradome sequencing

Additional file 8. Primers used in this study

The datasets of two small RNA libraries have been deposited to NCBI under GEO accession GSE83508 and will be available upon acceptance.

Additional file 1. Sodium distribution in 24 h 200 mM NaCl-treated ice plant seedlings. Fluorescence detection of intact root stained with 10 mM Sodium Green for 30 min. Roots of one-week-old seedlings were treated with MS medium (control) or MS medium plus 200 mM NaCl (salt) for 24 h. Images of Sodium Green fluorescence are showed on the left and bright field images are showed on the right. Bar = 1 mm.

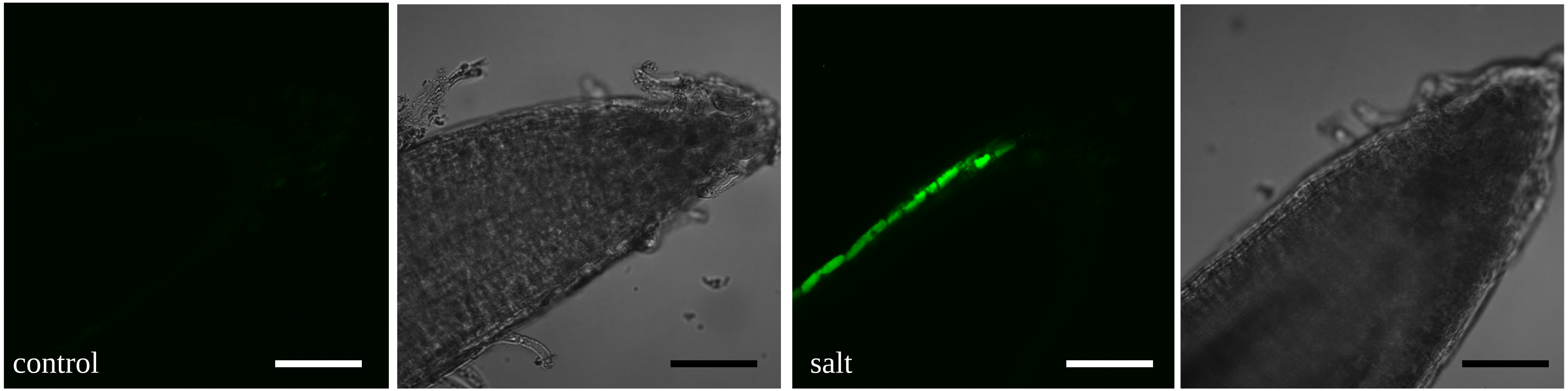

Additional file **2.** Summary of small RNA sequencing (A) and proportion distribution of 18-25 nt clean reads (B)

A

	Control		Salt	
Process	Redundant	Unique	Redundant	Unique
Total reads	5,215,338	1,449,046	5,080,508	1,483,730
Adaptors trimmed	5,126,125	1,303,617	5,052,296	1,327,609
Sequence outside 18-25 nt removed	3,827,104	1,126,686	3,708,379	1,137,337
Sequence contain unclear nucleotides removed	3,824,318	1,124,918	3,705,732	1,135,686
t/rRNA removed	3,089,326	1,106,388	3,157,610	1,118,714

B

	Control				Salt			
	Redundant		Unique		Redundant		Unique	
	Reads	Percentage	Reads	Percentage	Reads	Percentage	Reads	Percentage
Length 18	72,897	2.4	22,405	2.0	71,885	2.3	22,478	2.0
Length 19	316,373	10.2	29,870	2.7	323,951	10.3	30,197	2.7
Length 20	327,461	10.6	39,935	3.6	295,695	9.4	40,364	3.6
Length 21	682,125	22.1	64,928	5.9	752,906	23.8	65,683	5.9
Length 22	202,212	6.6	62,983	5.7	213,667	6.8	61,702	5.5
Length 23	336,275	10.9	220,873	20.0	332,044	10.5	220,736	19.7
Length 24	1,037,342	33.6	605,492	54.7	1,060,245	33.6	620,109	55.4
Length 25	114,641	3.7	59,902	5.4	107,217	3.4	57,445	5.1
Total	3,089,326	100	1,106,388	100	3,157,610	100	1,118,714	100

Additional file 3. Percentage of unique sequences starting with an A, C, G, and U in each size group in control (A) and salt (B) seedlings. A (red), C (green), G (yellow), and U (blue) percentage of unique sequences 5’ end in each size group.

A

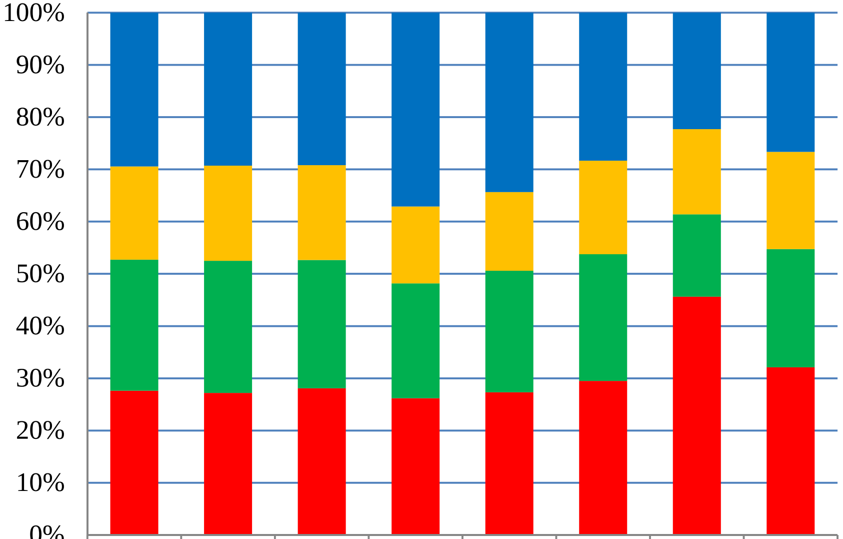

	18	19	20	21	22	23	24	25
U	29.45	29.27	29.19	37.09	34.34	28.32	22.3	26.64
G	17.83	18.23	18.16	14.73	15.06	17.91	16.3	18.62
C	25.09	25.3	24.57	22	23.28	24.27	15.78	22.62
A	27.64	27.2	28.08	26.17	27.32	29.5	45.62	32.11

B

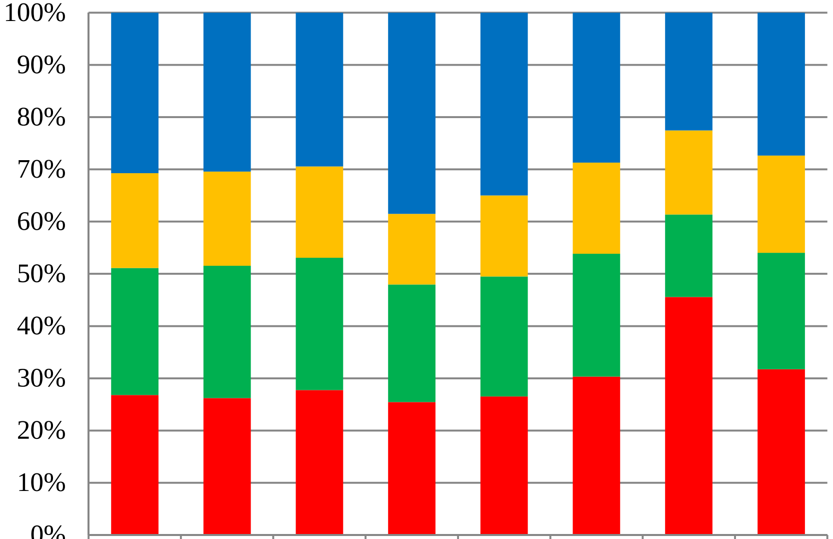

	18	19	20	21	22	23	24	25
U	30.73	30.41	29.42	38.5	34.99	28.69	22.52	27.36
G	18.15	18.04	17.49	13.53	15.52	17.46	16.12	18.62
C	24.32	25.33	25.34	22.51	22.98	23.53	15.8	22.3
A	26.8	26.22	27.75	25.45	26.51	30.32	45.56	31.72

Additional file 4. Summary of poly $(A)^+$ RNA sequencing in ice plant seedlings

Sequencing Summary

Run	Control	Salt
Total Reads	53,031,502	55,702,462
Read Length	101	101
Total Base	5356181702	5625948662
Reads after QT	51,573,704	54,966,600
Average Read Length after QT	96.5	98.6

QT = Quality Trim, criteria = min length = 35, error probability < 0.05

***De novo* Assembly**

Max contig length	20106
Min contig length	200
Avg contig length	1504.809
Total contigs length	35990520
N50	2125
Number of contig	23917
Number of contig >= 300b	21827
Total bases in contigs > 300	35477593
The percentage of contig length >= 300b	0.985748
Number of contig >= 1kb	14177
Total bases in contigs > 1k	30875263
The percentage of contig length >= 1kb	0.857872
Number of contig >= 40kb	0
Total bases in contigs > 40k	0
The percentage of contig length >= 40kb	0
Number of 'N'	0
N-ratio	0
Number of 'N' in >= 40kb contig	0

Mapping Statistics

Run	Control	Salt
Matched Reads	46,575,214	50,755,325
Total Matched Base	4,523,105,660	5,013,700,687
Reads mapped in pairs	41,946,510	47,025,644
Reads mapped in broken pairs	4,628,704	3,729,681
Not Matched Reads	3,663,816	3,523,111

Additional file 5**.** Sequences and length polymorphisms of conserved miRNAs in ice plant seedlings

miRNA fanily	Sequence	Length	Control read	Salt read	Control read (RPM)	Salt read (RPM)	Homolog	Target gene	Functions
miR156/157	**TGACAGAAGAGAGTGAGCAC**	20	18741	30883	6066	9780	zma-miR156i	*SPL3, SPL10, SPL11*	Flowering time control; phase changing modulation; later embryonic maturation and root development
	TTGACAGAAGAGAGTGAGCAC	21	3073	4516	995	1430	gma-miR156k		
	**CTGACAGAAGATAGAGAGCAC**	21	1796	1600	581	507	smo-miR156b		
	**TTGACAGAAGATAGAGAGCAC**	21	546	653	177	207	ath-miR157a		
	TGACAGAAGAGAGTGAGCACT	21	541	648	175	205	gma-miR156q		
	TGACAGAAGATAGAGAGCAC	20	71	87	23	28	ath-miR157d		
	CGACAGAAGAGAGTGAGCAC	20	48	72	16	23	ath-miR156g		
	GCTCTCTATGCTTCTGTCATC	21	38	64	12	20	aly-miR157d*		
	TGACAGAAGAGAGTGAGCACA	21	37	51	12	16	bna-miR156a		
	TGACAGAAGAGAGGGAGCAC	20	30	47	10	15	ptc-miR156k		
	TGACAGAAGAGAGCGAGCAC	20	27	32	9	10	zma-miR156k		
	**TGACAGAAGAGAGAGAGCAC**	20	24	39	8	12	ahy-miR156a		
	TGTCAGAAGAGAGTGAGCAC	20	21	31	7	10	ghr-miR156c		
	TGACAGAAGAGAGTGAGCATA	21	14	24	5	8	cca-miR156b		
	TGACAGAAGAGAGTGAGTAC	20	12	17	4	5	hci-miR156a		
	TTGACAGAAGAGAGAGAGCAC	21	10	22	3	7	ahy-miR15		
	TGACAGAAGAGAATGAGCAC	20	8	5	3	2	nta-miR156f		
	TGACAGAAGAAAGTGAGCAC	20	7	12	2	4	mdm-miR156ad		
	TGACAGAAGAGGGTGAGCAC	20	5	14	2	4	mtr-miR156j		
	TGACAGAGGAGAGTGAGCAC	20	5	9	2	3	vvi-miR156e		
	GCTCACTCTCTATCTGTCACC	21	2	0	1	0	aly-miR156f*		
	TTGACAGAAGATGGAGAGCAC	21	2	2	1	1	ptc-miR156l		
	GCTCACTGCTCTATCTGTCACC	22	1	0	0	0	zma-miR156l*		
	TTGACAGAAGAAAGAGAGCAC	21	1	1	0	0	smo-miR156c		
	TTGACAGAAGATAGAGGGCAC	21	1	0	0	0	mtr-miR156g		
	CTGACAGAAGATAGAGAGCAT	21	0	2	0	1	gma-miR156r		
	TGACAGAAGAGAGAGAGCACA	21	0	1	0	0	osa-miR156k		

miR159	**TTTGGATTGAAGGGAGCTCC**	20	11545	12186	3737	3859	aly-miR159c	*MYB33, MYB65, MYB101*	Flowering time control in short-day photoperiods; leaf development; seed size and shape determination
	**TTTGGATTGAAGGGAGCTCTA**	21	4319	3594	1398	1138	ath-miR159a		
	TTTGGATTGAAGGGAGCTCCT	21	58	45	19	14	ath-miR159c		
	TTTGGATTGAAGGGAGCTCTT	21	42	32	14	10	ath-miR159b		
	ATTGGATTGAAGGGAGCTCC	20	17	20	6	6	osa-miR159c		
	TTTGGTTTGAAGGGAGCTCTA	21	8	3	3	1	pde-miR159		
	CTTGGATTGAAGGGAGCTCC	20	5	9	2	3	mdm-miR159a		
	CTTGGATTGAAGGGAGCTCTA	21	5	3	2	1	osa-miR159f		
	TTTGGATTGAAGGGAGCTCTG	21	2	4	1	1	osa-miR159a.1		
	CTTGGATTGAAGGGAGCTCCC	21	1	1	0	0	pta-miR159c		
	TTTGGACTGAAGGGAGCTCTA	21	1	1	0	0	aqc-miR159		
miR160	**TGCCTGGCTCCCTGTATGCCA**	21	4	3	1	1	ath-miR160a	*ARF10, ARF16, ARF17*	Root cap structure and lateral root development; seed development during embryogenesis; flower and leaf development
	GCGTATGAGGAGCCAAGCATA	21	1	0	0	0	gma-miR160a-3p		
miR162	**TCGATAAACCTCTGCATCCAG**	21	369	442	119	140	ath-miR162a	*DCL1*	Regulation of somatic embryogenesis, environmental stress response and fiber differentiation and development
	TCGATAAACCTCTGCATCCA	20	27	21	9	7	zma-miR162		
	GGAGGCAGCGGTTCATCGATC	21	1	1	0	0	aly-miR162a*		
	TCGATAAACCTCTGCATCCGG	21	1	1	0	0	bdi-miR162		
	TCGATAAACCTGTGCATCCAG	21	1	0	0	0	bna-miR162a		
miR164	**TGGAGAAGCAGGGCACGTGCA**	21	6	3	2	1	ath-miR164a	*CUC1, CUC2, NAC1*	Petal number control; development of leaf and shoot buds; lateral root development; normal embryonic development
	TGGAGAAGCAGGGCACGTGC	20	1	0	0	0	gma-miR164c		
	TCGGACCAGGCTTCATCCCCC	21	65	69	21	22	ath-miR165a		
	TCGGACCAGGCTTCATCCCC	20	14	16	5	5	aly-miR165a		
	**TCGGACCAGGCTTCATTCCCC**	21	169593	213009	54896	67459	ptc-miR166i		
	**TCTCGGACCAGGCTTCATTCC**	21	158432	179038	51284	56700	bdi-miR166f		
	TCGGACCAGGCTTCATTCCTC	21	3489	3370	1129	1067	osa-miR166g-3p		

miR165/166	CTCGGACCAGGCTTCATTCCC	21	1465	1032	474	327	bdi-miR166e	*HD-ZIP III*	Embryo development; leaf primordial, shoot apical meristem, and floral stem cell development; root and nodule development
	TCGGACCAGGCTTCATTCCC	20	701	774	227	245	sbi-miR166h		
	TCCGGACCAGGCTTCATTCCC	21	373	434	121	137	ppt-miR166j		
	TCGGACCAGGCTTCATTCCCCC	22	240	309	78	98	ctr-miR166		
	TCTCGGACCAGGCTTCATTC	20	211	246	68	78	gma-miR166u		
	TCGAACCAGGCTTCATTCCCC	21	179	226	58	72	osa-miR166e-3p		
	TCGGACCAGGCTTCATTCCCT	21	120	119	39	38	osa-miR166m		
	TCGGACCAGGCTTCATTCCCG	21	88	92	28	29	gma-miR166j-3p		
	TCGGACCAGGCTTCATTCCT	20	65	63	21	20	sbi-miR166k		
	TCGGACCAGGCTTCATTCCTA	21	4	1	1	0	mtr-miR166b		
	TCGGACCAGGCTTCATTCCTT	21	4	2	1	1	ptc-miR166n		
	TCGGACCAGGCTTCATTCTC	20	3	2	1	1	cme-miR166i		
	GGAATGTTGTCTGGCTCGAGG	21	2	0	1	0	zma-miR166c*		
	GGTTTGTTGTCTGGCTCGAGG	21	2	1	1	0	osa-miR166k-5p		
	GGATTGTTGTCTGGCTCGGGG	21	1	1	0	0	zma-miR166k*		
	TCGGATCAGGCTTCATTCCTC	21	1	1	0	0	osa-miR166j-3p		
	TCGGACCAGGCTCCATTCCTT	21	0	1	0	0	ptc-miR166p		
miR167	TGAAGCTGCCAGCATGATCTG	21	3903	3772	1263	1195	osa-miR167d-5p	*ARF6, ARF8*	Gynoecium and stamen maturation; seed development
	TGAAGCTGCCAGCATGATCTGA	22	544	624	176	198	ccl-miR167a		
	TGAAGCTGCCAGCATGATCTC	21	342	216	111	68	vvi-miR167c		
	TGAAGCTGCCAGCATGATCTA	21	8	10	3	3	ath-miR167a		
	TGAAGCTGCCAACATGATCTG	21	6	5	2	2	ptc-miR167h-5p		
	TGAAGCTGCCAGCATGATCTT	21	2	2	1	1	ptc-miR167f-5p		
	TGAAGCTGCCAGCATGATCTGG	22	1	4	0	1	ath-miR167d		
	GGTCATGCTCTGACAGCCTCAC	22	0	1	0	0	aly-miR167b*		
miR168	**TCGCTTGGTGCAGGTCGGGAA**	21	239	229	77	73	ath-miR168a	*AGO1*	Stress response and signal transduction in plant development
	TCGCTTGGTGCAGGTCGGGA	20	61	83	20	26	cme-miR168		
	CCCGCCTTGCATCAACTGAAT	21	21	37	7	12	aly-miR168a*		
	TCGCTTGGTGCAGGTCGGGAC	21	9	2	3	1	nta-miR168a		
	TCGCTTGGTGCAGGTCGAGAA	21	1	0	0	0	bna-miR168b		

miR169	**TAGCCAAGGATGACTTGCCT**	20	358	476	116	151	sbi-miR169r-5p	*HAP2-1*	Nodule development control
	**CAGCCAAGGATGACTTGCCGG**	21	4	5	1	2	ath-miR169b		
	GGCAAGTTGTCCTTGGCTACA	21	1	0	0	0	zma-miR169r*		
	TAGCCAAGGATGACTTGCCTG	21	1	2	0	1	ath-miR169h		
miR171	**TGATTGAGCCGTGCCAATATC**	21	141	118	46	37	osa-miR171b	*SCL6-II, SCL6-III, SCL6-IV, NSP2*	Negatively regulation on shoot branching; root colonization regulation
	TTGAGCCGCGCCAATATCACT	21	39	35	13	11	vvi-miR171f		
	TTGAGCCGTGCCAATATCACG	21	7	8	2	3	ath-miR171b		
	TGATTGAGCCGTGCCAATAT	20	2	0	1	0	htu-miR171a		
	TTGAGCCGTGCCAATATCAC	20	1	0	0	0	zma-miR171b-3p		
	TTGAGCCGTGCCAATATCACT	21	1	0	0	0	smo-miR171a		
miR172	AGAATCTTGATGATGCTGCAG	21	2	1	1	0	ath-miR172c	*AP2, AG, gl15*	The onset of flowering; seed development; phase change in shoot
	AGAATCTTGATGATGCTGCA	20	0	2	0	1	zma-miR172c-3p		
	GCAGCACCATCAAGATTCACA	21	0	1	0	0	aly-miR172b*		
miR319	**TTGGACTGAAGGGAGCTCCCT**	21	4113	3571	1331	1131	ath-miR319a	*TCPs*	Leaf morphogenesis, complexity and senescence; flower development; male and female gametophyte development
	TTGGACTGAAGGGAGCTCCC	20	1098	1249	355	396	mtr-miR319a		
	CTTGGACTGAAGGGAGCTCCC	21	767	857	248	271	ppt-miR319c		
	CTTGGACTGAAGGGAGCTCC	20	27	33	9	10	ppt-miR319a		
	TTGGACTGAAGGGAGCTCCT	20	11	7	4	2	ptc-miR319e		
	TTGGACTGAAGGGAGCTCCCA	21	8	12	3	4	vvi-miR319g		
	TTGGACTGAAGGGAGCTCCTT	21	4	2	1	1	ath-miR319c		
	TTGGACTGAAGGGTGCTCCCT	21	3	1	1	0	bdi-miR319b		
	CTTGGACTGAAGGGAGCTCCT	21	2	4	1	1	vun-miR319b		
	TTGGGCTGAAGGGAGCTCCC	20	1	0	0	0	ptc-miR319i		
miR390	AAGCTCAGGAGGGATAGCGCC	21	129	109	42	35	ath-miR390a	*TAS3*	Lateral root development
	AAGCTCAGGAGGGATAGCACC	21	1	0	0	0	gma-miR390b		
miR393	TCCAAAGGGATCGCATTGATCC	22	2	3	1	1	ath-miR393a	*TAAR*	Auxin-related leaf, root and shoot development
	TCCAAAGGGATCGCATTGATC	21	1	4	0	1	osa-miR393a		
	TCCAAAGGGATCGCATTGATCT	22	1	4	0	1	osa-miR393b-5p		
	ATCATGCTATCCCTTTGGATT	21	0	1	0	0	ptc-miR393b-3p		

miR394	TTGGCATTCTGTCCACCTCC	20	41	22	13	7	ath-miR394a	*LCR*	Regulation of leaf curling-related morphology
miR396	TTCCACAGCTTTCTTGAACTT	21	3244	4983	1050	1578	ath-miR396b	*GRFs*	Cell proliferation control during leaf and root development
	**TTCCACAGCTTTCTTGAACTG**	21	1189	1354	385	429	ath-miR396a		
	GTTCAAGAAAGCTGTGGGAAA	21	193	140	62	44	cca-miR396a-3p		
	TTCCACAGCTTTCTTGAACT	20	120	195	39	62	vvi-miR396b		
	GTTCAATAAAGCTGTGGGAAG	21	77	69	25	22	aly-miR396a*		
	TTCCACAGCTTTCTTGAACTGT	22	9	14	3	4	gma-miR396e		
	TTCAAGAAAGCTGTGGGAAAA	21	8	4	3	1	cca-miR396c		
	TTCCACAGCTTTCTTGAACTA	21	6	8	2	3	vvi-miR396a		
	GTTCAATAAAGCTGTGGGAA	20	4	9	1	3	osa-miR396a-3p		
	TTCAATAAAGCTGTGGGAAG	20	3	7	1	2	gma-miR396a-3p		
	GTTCAATAAAGCTGTGGGAAA	21	1	0	0	0	zma-miR396b*		
	TTCCACGGCTTTCTTGAACTG	21	0	2	0	1	ptc-miR396f		
miR397	TCATTGAGTGCAGCGTTGATG	21	2	2	1	1	ath-miR397a	*L-AO*	Seed development in rice
miR398	TGTGTTCTCAGGTCGCCCCTG	21	218	307	71	97	osa-miR398b	*CSD1; CSD2; CCS1*	Regulation of oxidative stress and copper homeostasis
	TGTGTTCTCAGGTCACCCCTG	21	1	0	0	0	ath-miR398b		
	TGTGTTCTCAGGTCGCCCCCG	21	1	1	0	0	zma-miR398a		
miR399	TGCCAAAGGAGAATTGCCCTG	21	21	10	7	3	osa-miR399a	*PHO2, UBC*	Regulation of cellular response to local Pi increase during arbuscular mycorrhizal symbiosis
	CGCCAAAGGAGAATTGCCCTG	21	1	0	0	0	tcc-miR399e		
	TGCCAAAGGAGAATTGCCCGG	21	1	0	0	0	ssl-miR399		
miR403	**TTAGATTCACGCACAAACTCG**	21	3654	3511	1183	1112	ath-miR403	*AGO2*	Stress response and signal transduction in plant development
	TTAGATTCACGCACAAACTTG	21	17	15	6	5	gma-miR403a		
miR408	TGCACTGCCTCTTCCCTGGCT	21	16	23	5	7	ppt-miR408b	*Cupredoxin*	Root development under many stress conditions
	TGCACTGCCTCTTCCCTGGC	20	1	0	0	0	gma-miR408d		

Boldface indicates the sequence matched to ice plant transcriptome and has predicted hairpin structure

Additional file 6. Predicted hairpin structure of conserved mcr-miRNA precursors. Secondary structure prediction was carried out with mfold. miRNAs were labeled with red dots and miRNA*s with blue dots.

mcr-miR156 cand 1

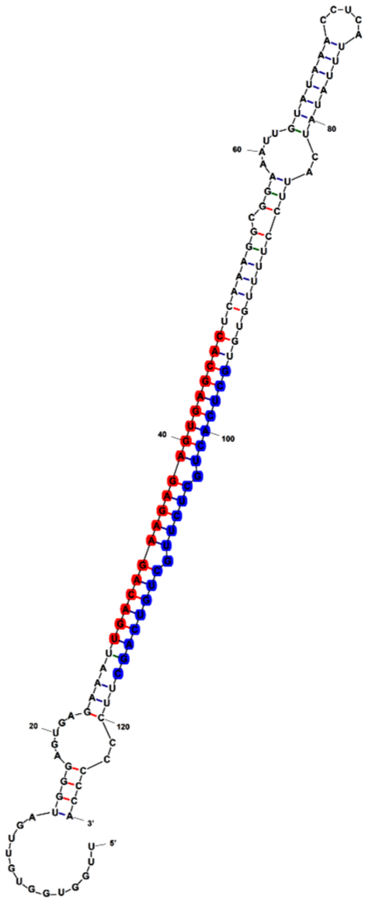

mcr-miR156 cand 2

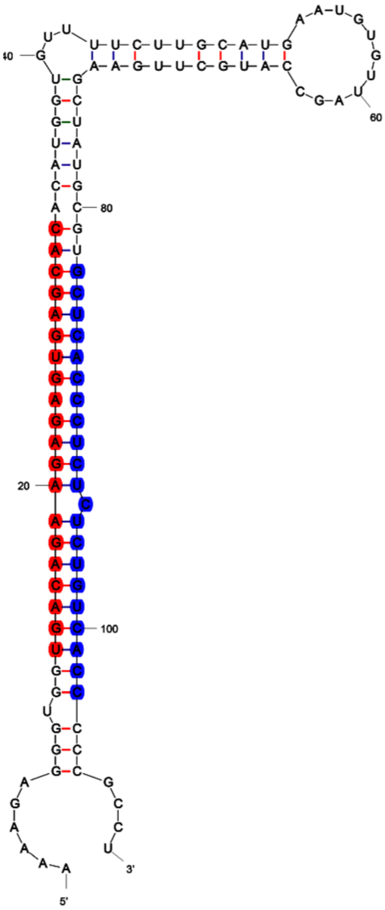

mcr-miR156 cand 3

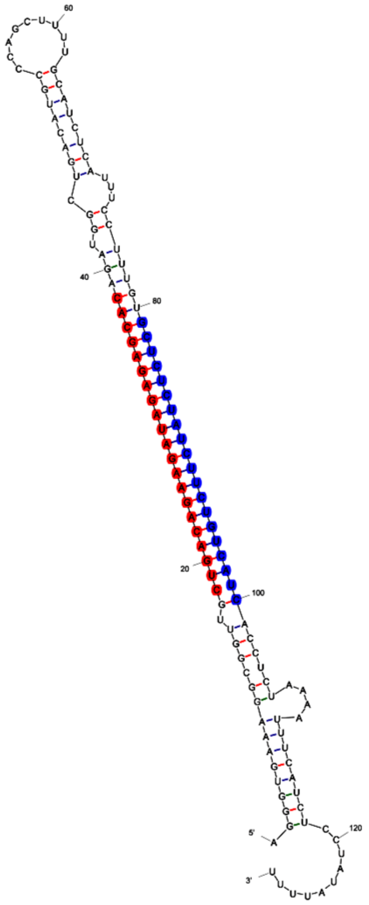

mcr-miR156 cand 4

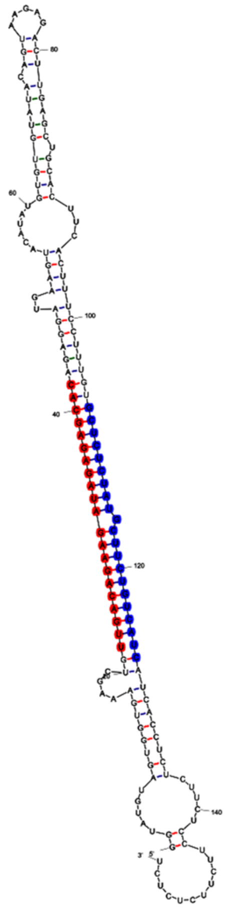

mcr-miR156 cand 5

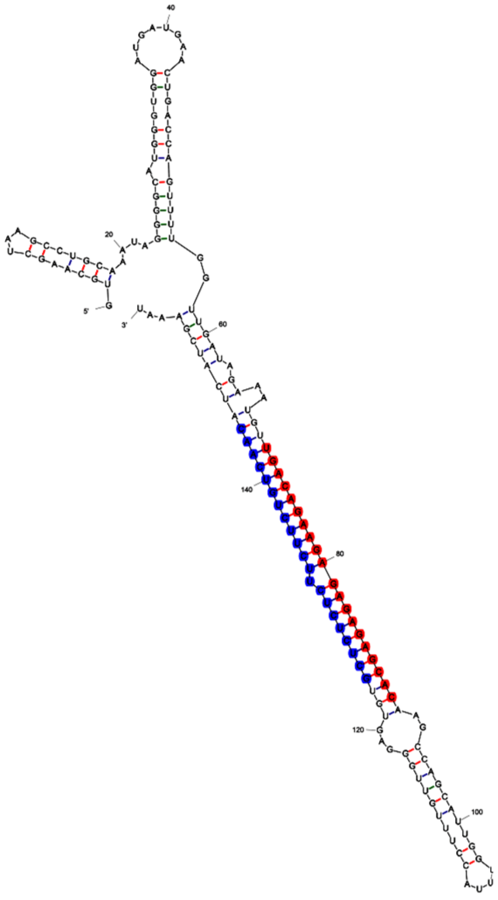

mcr-miR160 candidate

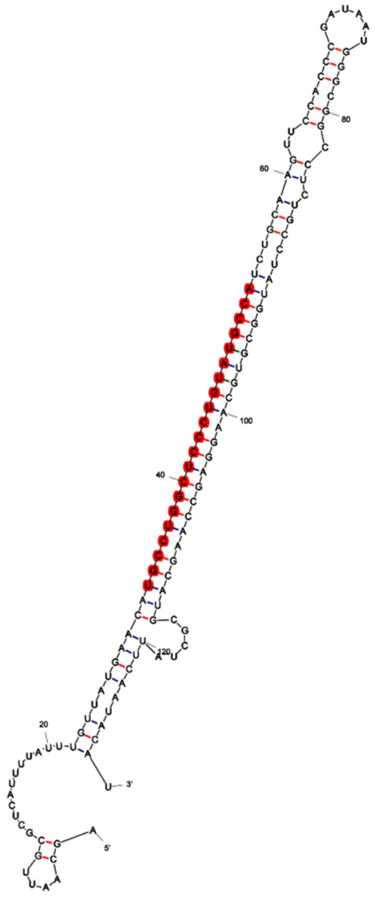

Additional file 6. Predicted hairpin structure of conserved mcr-miRNA precursors (continued)

mcr-miR162 candidate

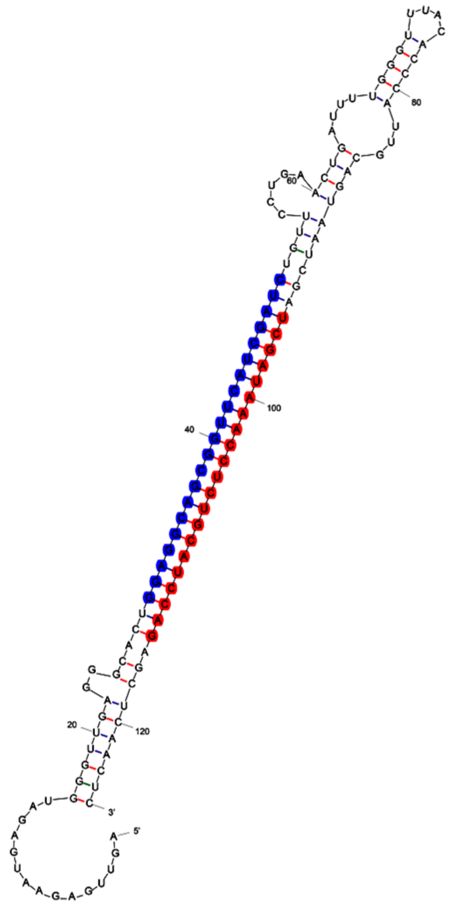

mcr-miR166 cand 1

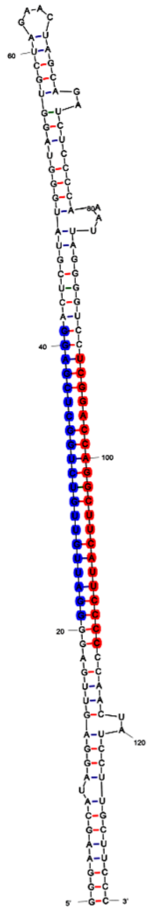

mcr–miR169 cand 1

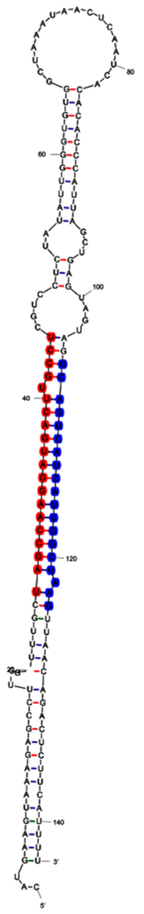

mcr-miR169 cand 2

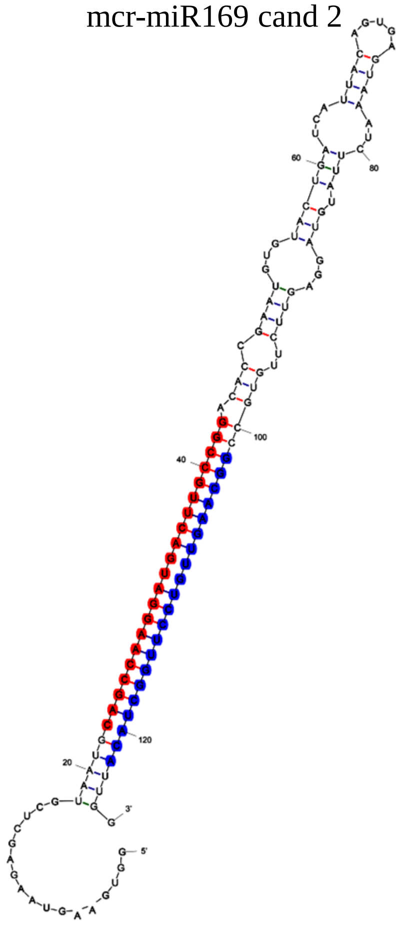

mcr-miR171 candidate

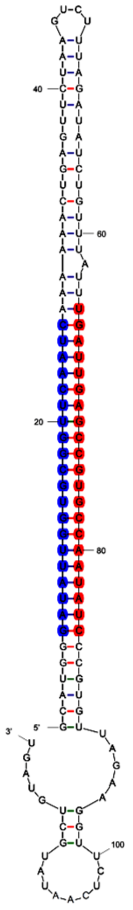

mcr-miR403 candidate

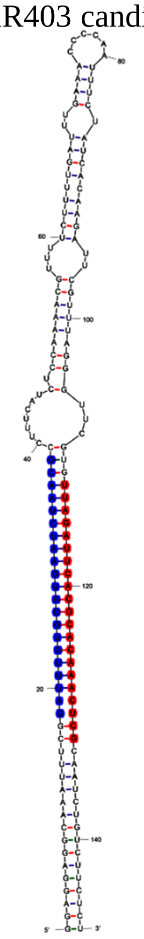

Additional file 6. Predicted hairpin structure of conserved mcr-miRNA precursors (continued)

mcr-miR159 cand 1

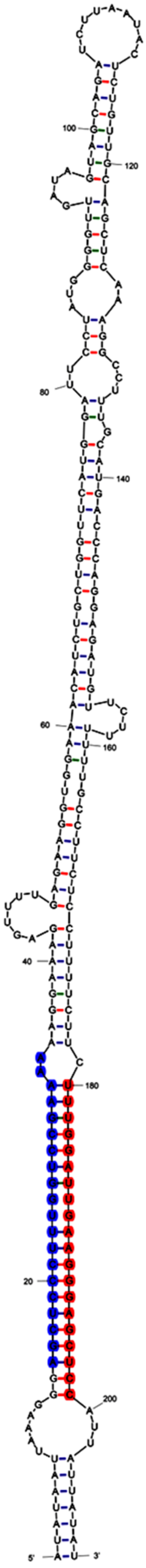

mcr-miR159 cand 2

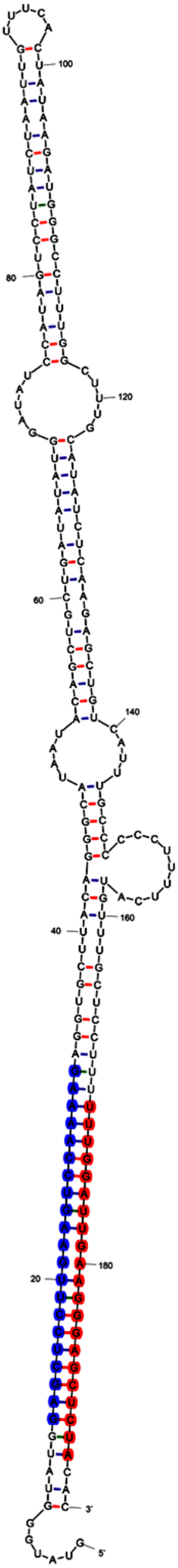

mcr-miR166 cand 2

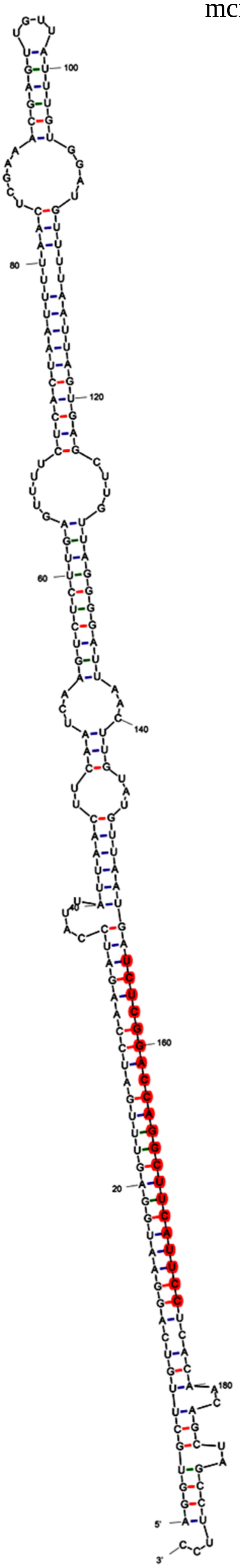

mcr-miR168 candidate

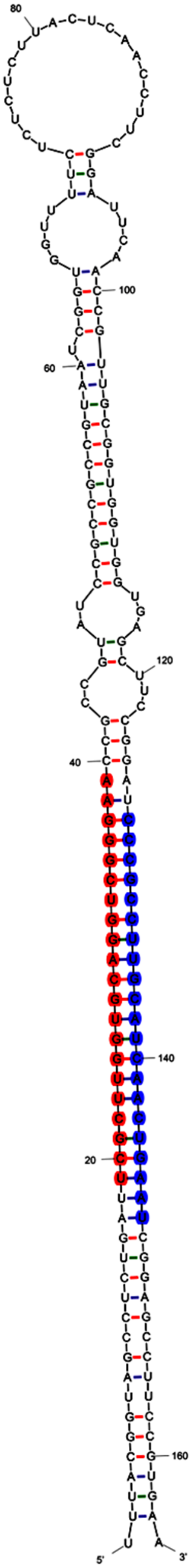

mcr-miR396 candidate

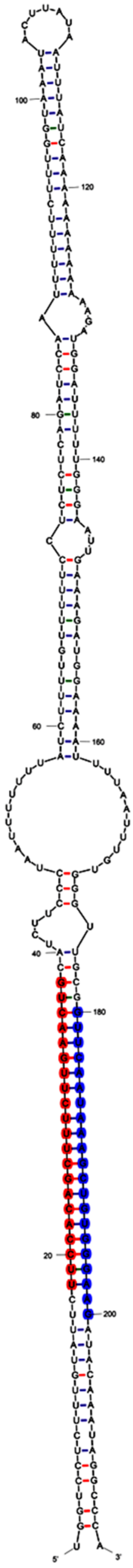

Additional file 6. Predicted hairpin structure of conserved mcr-miRNA precursors (continued)

mcr-miR164 candidate

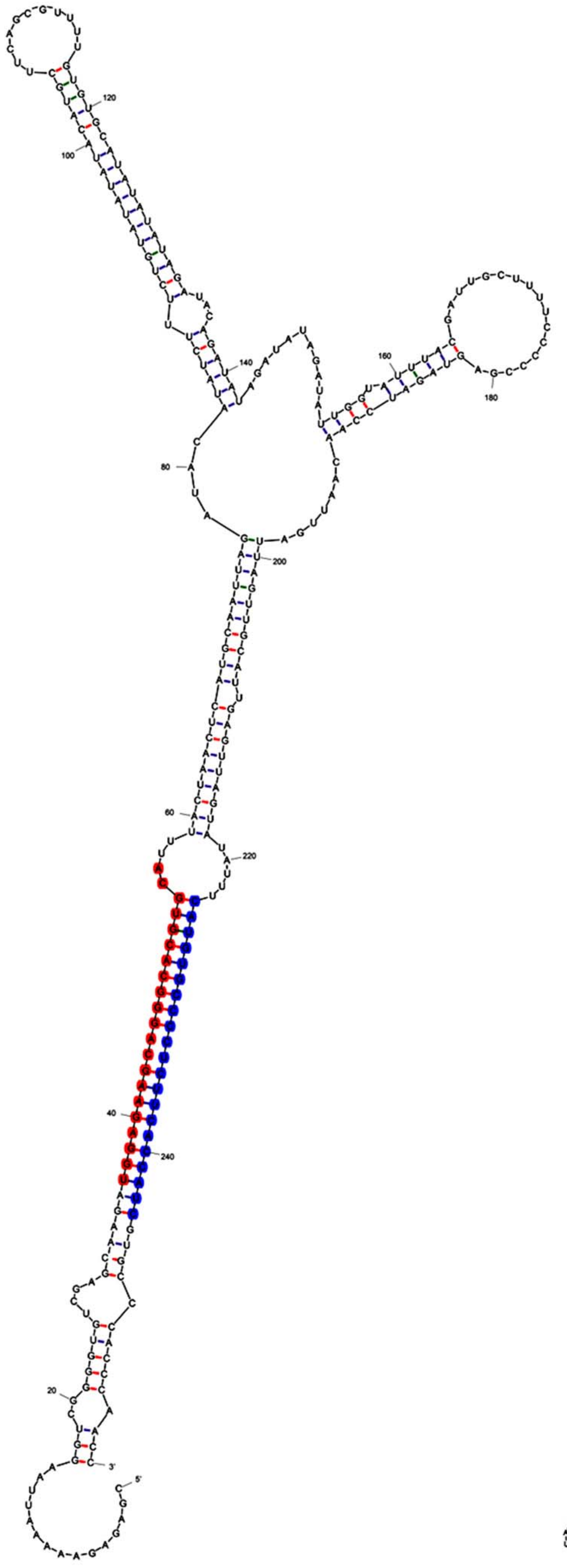

mcr-miR319 candidate

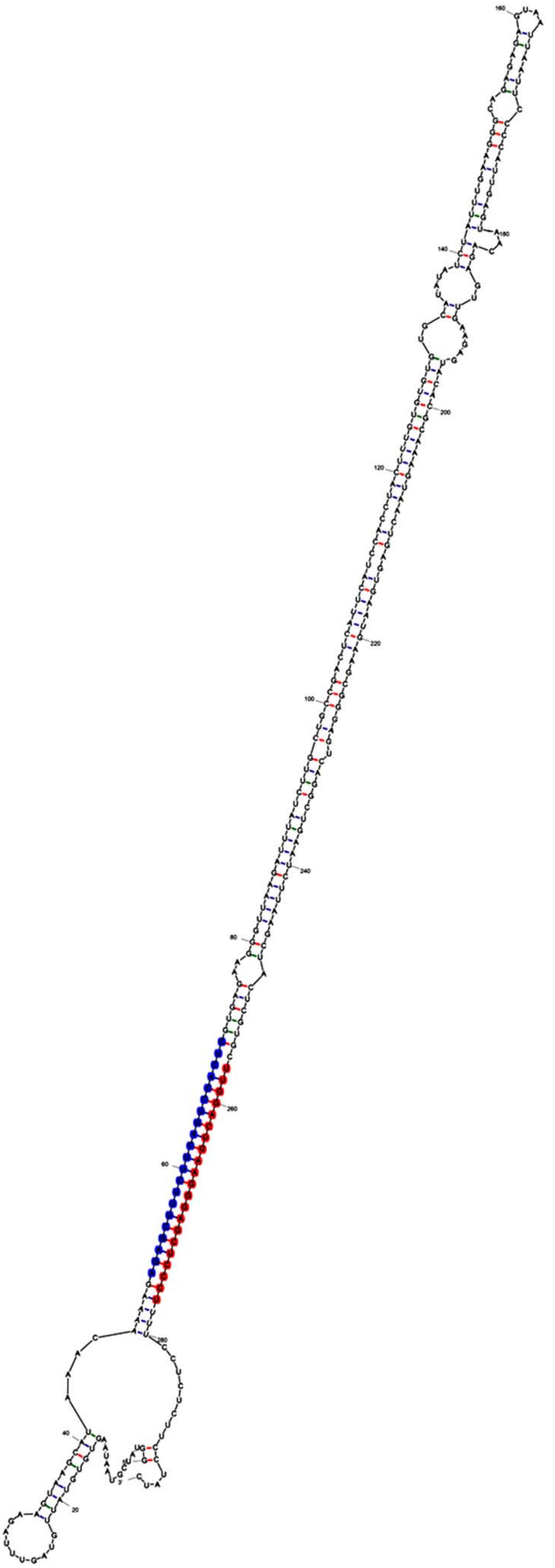

Additional file 6. Predicted hairpin structure of novel mcr-miRNA precursors. Secondary structure prediction was carried out with mfold. miRNAs were labeled with red dots and miRNA*s with blue dots.

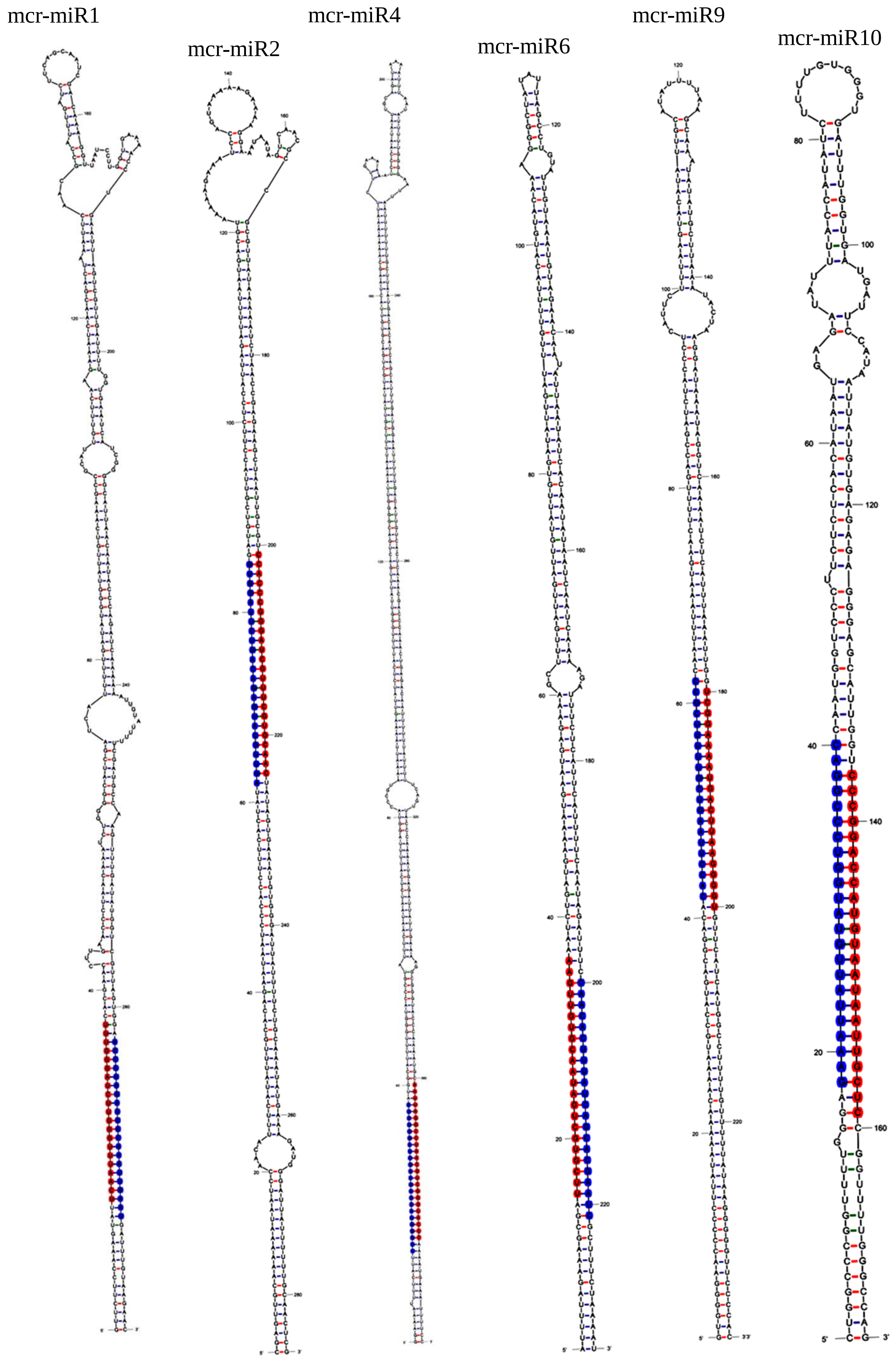

Additional file 6. Predicted hairpin structure of novel mcr-miRNA precursors (continued)

mcr-miR3

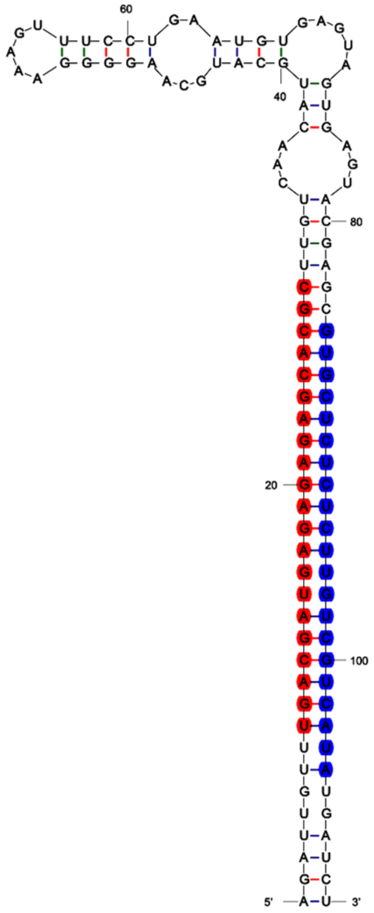

mcr-miR5

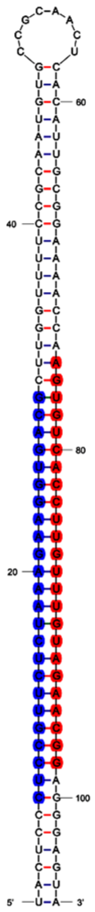

mcr-miR7

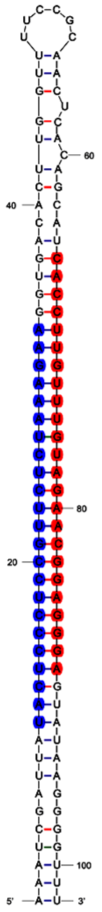

mcr-miR8

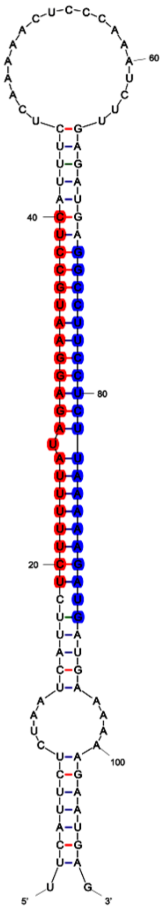

mcr-miR11

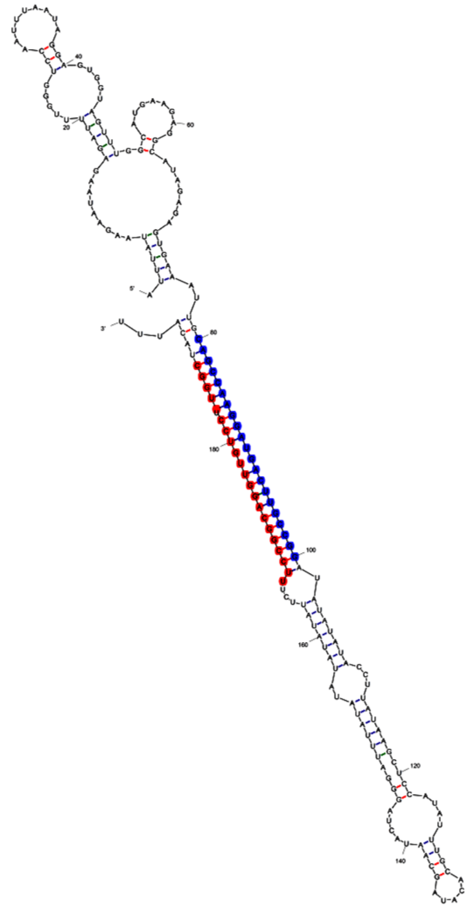

mcr-miR15

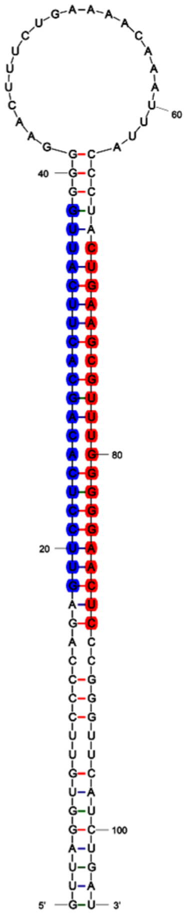

mcr-miR19

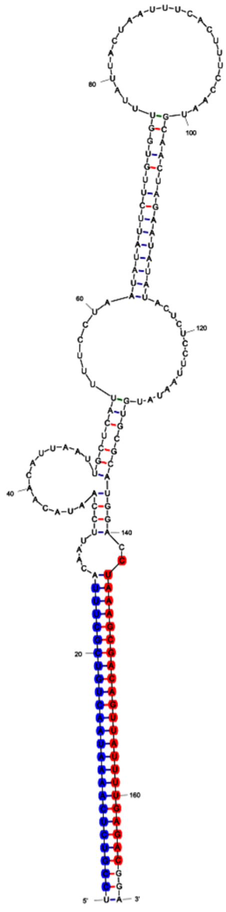

mcr-miR24

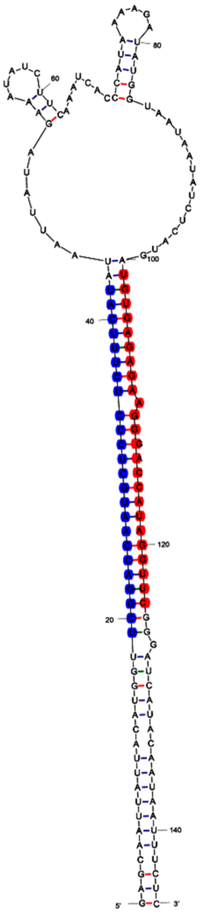

Additional file 6. Predicted hairpin structure of novel mcr-miRNA precursors (continued)

mcr-miR12

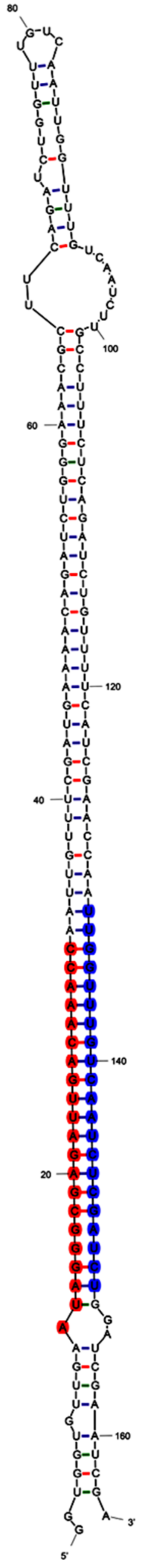

mcr-miR13

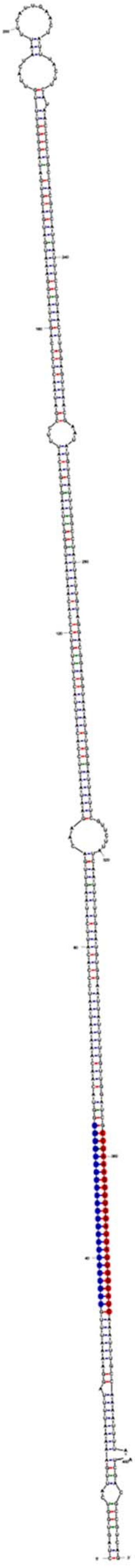

mcr-miR14

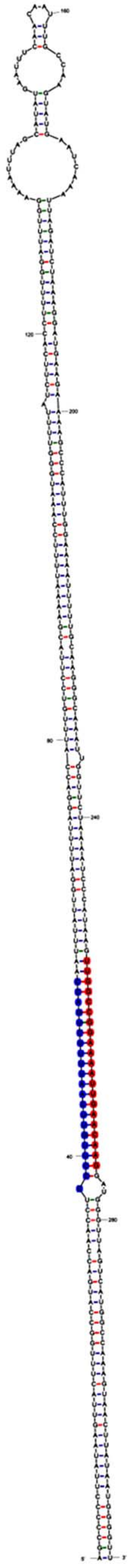

mcr-miR16

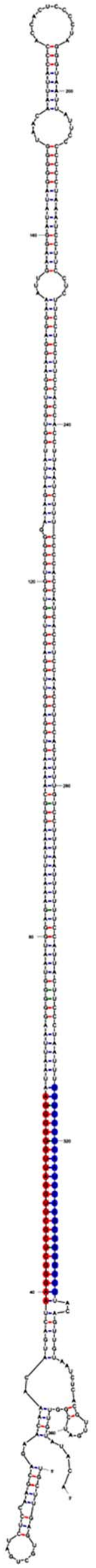

mcr-miR17

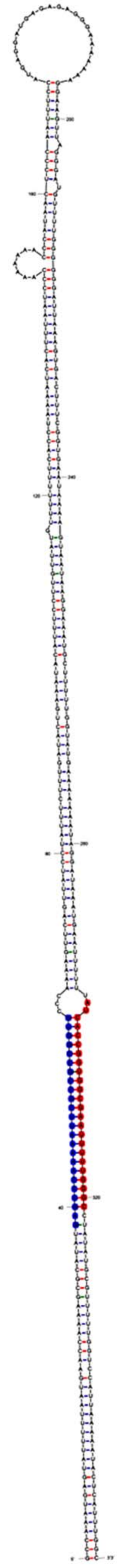

Additional file 6. Predicted hairpin structure of novel mcr-miRNA precursors (continued)

mcr-miR18

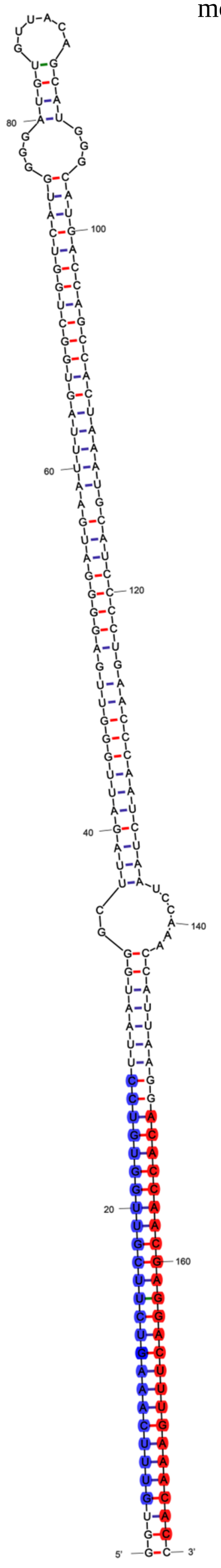

mcr-miR20

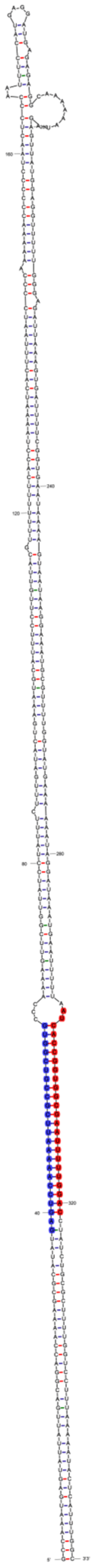

mcr-miR21

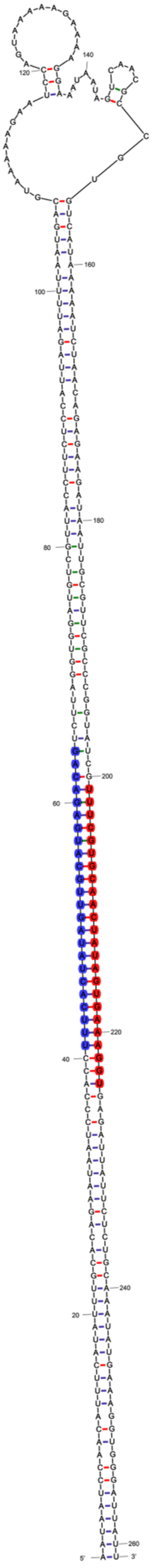

mcr-miR22

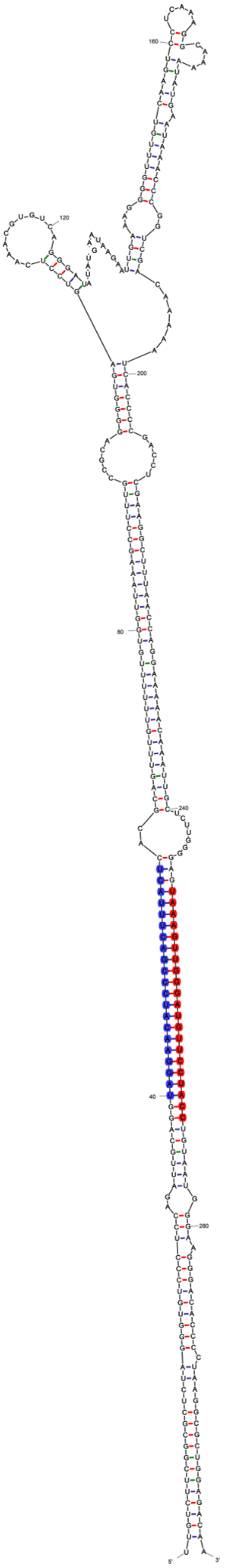

mcr-miR23

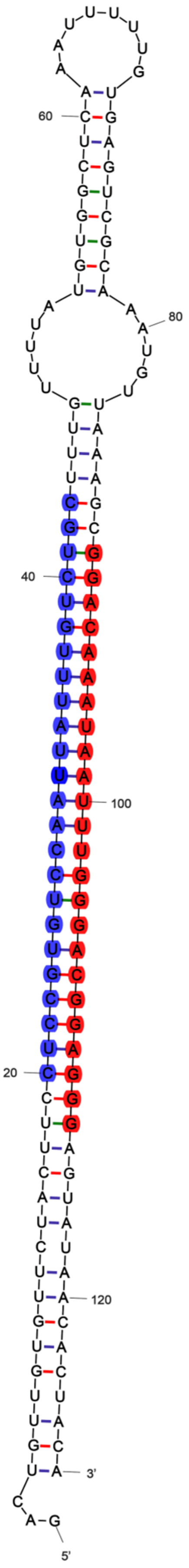

Additional file 7. Target plots (t-plots) show mcr-miR164 (A), 166b (B), 169a, b (C), and 403 (D) targeting to corresponding genes using degradome sequencing. Arrows indicate the miRNA cleavage sites.

A mcr-miR164 targeting gene coding NAC100

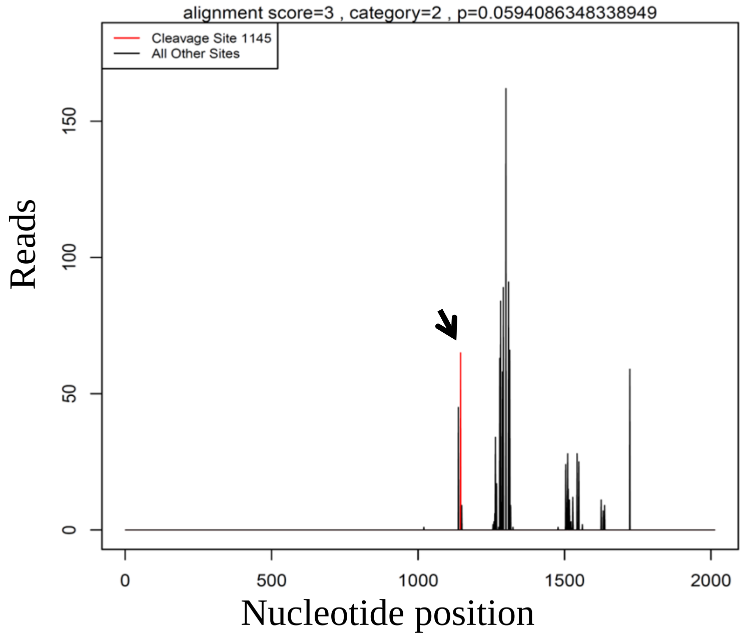

B mcr-miR166b targeting gene coding HD-ZIP

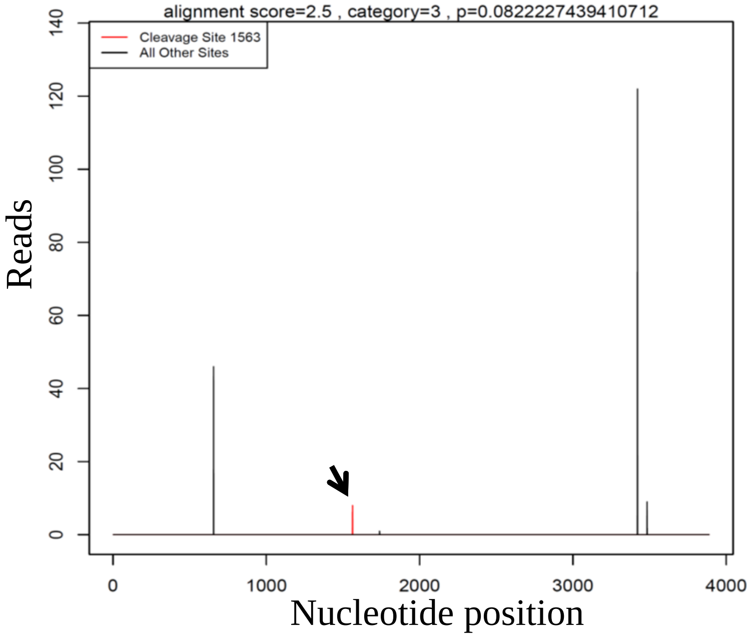

C mcr-miR169a, b targeting gene coding NF-YA1

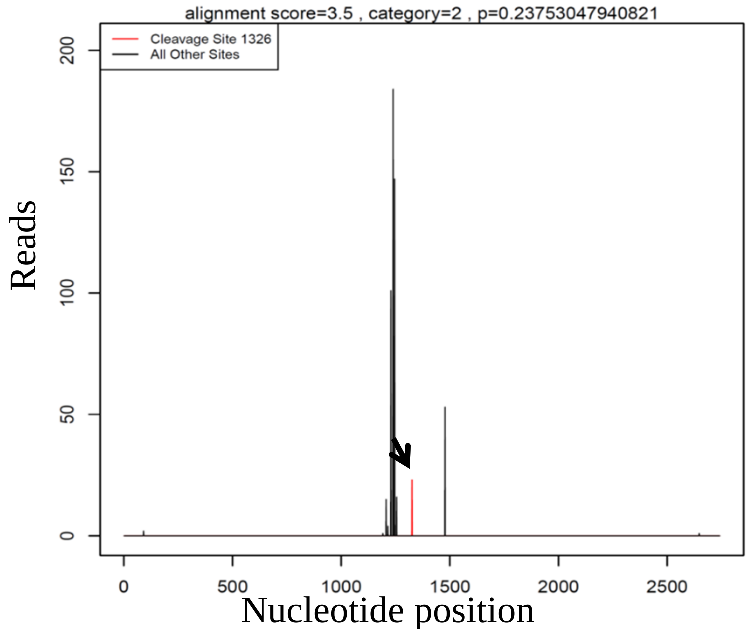

D mcr-miR403 targeting gene coding AGO2

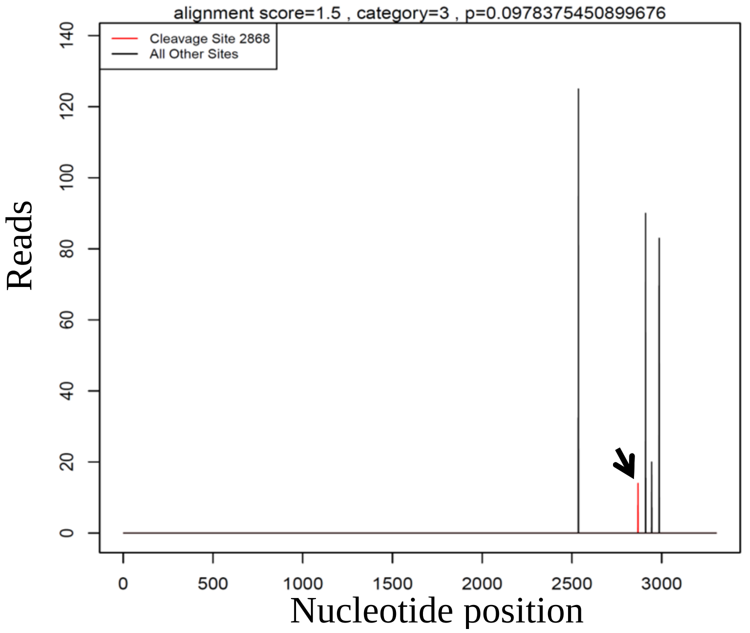

Additional file 8. Primers used in this study. SL: stem-loop; PA: poly A; F: forward primer; R: reverse primer

RT primers	Sequence
SLRT-156a	GTTGGCTCTGTCTAAACATTATAGCGCGTAGTTAGACAGAGCCAACGTGCTC
SLRT-159b	GTTGGCTCTGTCTAAACATTATAGCGCGTAGTTAGACAGAGCCAACTAGAGC
SLRT-160	GTTGGCTCTGTCTAAACATTATAGCGCGTAGTTAGACAGAGCCAACTGGCAT
SLRT-162	GTTGGCTCTGTCTAAACATTATAGCGCGTAGTTAGACAGAGCCAACCTGGAT
SLRT-164	GTTGGCTCTGTCTAAACATTATAGCGCGTAGTTAGACAGAGCCAACTGCACG
SLRT-166b	GTTGGCTCTGTCTAAACATTATAGCGCGTAGTTAGACAGAGCCAACGGAATG
SLRT-168	GTTGGCTCTGTCTAAACATTATAGCGCGTAGTTAGACAGAGCCAACTTCCCG
SLRT-169b	GTTGGCTCTGTCTAAACATTATAGCGCGTAGTTAGACAGAGCCAACCCGGCA
SLRT-171	GTTGGCTCTGTCTAAACATTATAGCGCGTAGTTAGACAGAGCCAACGATATT
SLRT-319	GTTGGCTCTGTCTAAACATTATAGCGCGTAGTTAGACAGAGCCAACAGGGAG
SLRT-396	GTTGGCTCTGTCTAAACATTATAGCGCGTAGTTAGACAGAGCCAACCAGTTC
SLRT-403	GTTGGCTCTGTCTAAACATTATAGCGCGTAGTTAGACAGAGCCAACCGAGTT
SLRT-R1	GTTGGCTCTGTCTAAACATTATAGCGCGTAGTTAGACAGAGCCAACAAACCC
SLRT-R3	GTTGGCTCTGTCTAAACATTATAGCGCGTAGTTAGACAGAGCCAACGCGTGC
SLRT-R5	GTTGGCTCTGTCTAAACATTATAGCGCGTAGTTAGACAGAGCCAACCCGTTC
Universal-PART	ACATTATAGCGCGTAGTTAGACTAAGATATGTAGAGAGCCAGATTTTTTTTTTTTTTVN

miRNA-specific PCR primers	Sequence	Application
156-PAF	TGACAGAAGAGAGTGAGCAC	Poly(A)
156-SLF	TGACAGAAGAGAGTGAG	Stem-Loop
159-F	TTCTTTGGATTGAAGGGAGC	Poly(A); Stem-Loop
160-PAF	ATGCCTGGCTCCCTGTATGC	Poly(A)
160-SLF	ATGCCTGGCTCCCTGTAT	Stem-Loop
162-F	AATCGATCGATAAACCTCTG	Poly(A); Stem-Loop
164-F	AGCAAGATGGAGAAGCAGGG	Poly(A); Stem-Loop
166-PAF	TCTCGGACCAGGCTTCATTC	Poly(A)
166-SLF	TCTCGGACCAGGCTTCAT	Stem-Loop
168-PAF	TTCGCTTGGTGCAGGTCGGG	Poly(A)
168-SLF	TTCGCTTGGTGCAGGTCG	Stem-Loop
169-SLF	GCTAGCCAAGGATGACTTGC	Stem-Loop
169-PAF	TTGCTAGCCAAGGATGACTT	Poly(A)
171-SLF	TTTGATTGAGCCGTGCCAAT	Stem-Loop
171-PAF	TATTTGATTGAGCCGTGCCA	Poly(A)
319-F	TGTCGTTGGACTGAAGGGAG	Poly(A); Stem-Loop
396-PAF	CTTCCACAGCTTTCTTGAAC	Poly(A)
396-SLF	CTTCCACAGCTTTCTTGAA	Stem-Loop
403-F	TGTTAGATTCACGCACAAAC	Poly(A); Stem-Loop
R1-F	AAGTATTCAATTTGGGTTCT	Poly(A); Stem-Loop
R2-F	CCACCCGGGATCGTTTCGTG	Poly(A)
R3-F	TTGACGATGAGAGAGAGCAC	Poly(A); Stem-Loop

R4-F	TTAGGCCTAACGTCGGGTAC	Poly(A)
R5-F	AGTGTCACCTTGTTTGTAGA	Poly(A); Stem-Loop
R6-F	AAGCGATTCGTGCTGATAAC	Poly(A)
R7-F	CATCACCTTGTTTGTAGAAC	Poly(A)
R8-F	ATGTCTTTTTATAGAGGAAT	Poly(A)
R9-F	ATTGGTCGGAAAATGACTTA	Poly(A)
R10-F	GTCCCGGACCATGTAATAAT	Poly(A)
R11-F	ATTCTTTCCGGCAGGTTGTC	Poly(A)
R12-F	TGAATAGGGCGAGATTGACA	Poly(A)
U6-F	GATAAAATTGGAACGATACAG	Poly(A); Stem-Loop
U6-R	ATTTGGACCATTTCTCGATTT	Poly(A); Stem-Loop
Univeral-R	ACATTATAGCGCGTAGTTAGA	Poly(A); Stem-Loop

Target gene primers	Sequence	Region
SPL-F	TCAACCGTTCAAGGATTACC	Target site
SPL-R	CATTCTCAAGCACTGGCATAG	
MYB-F	CGCAGAAGAAGAAGCTCGTATCA	Conserve region
MYB-R	TGTCAAAATCATTCGCCTGTG	
ARF-F	TTCCCATGGTGAGCAAACAA	Target site
ARF-R	CTTCCAGTCCCCTCGTATTCC	
DCL-F	GGATGCTAGCTGGCTTTCTGTAA	Target site
DCL-R	GGGGATTGGATTTTGTCTGGA	
NAC-F	CATTCGGTAACGAGTTTAGCCCTTC	Target site
NAC-R	GCCCTAGAGAAATAGTCCCCTTGGT	
HD-ZIP-F	GCTCCTGCCCGTGATTTTTG	Conserve region
HD-ZIP-R	GCGCCAGCACAGTTGATGATT	
AGO1-F	CCGGAAAGGGTAGCACTG	Conserve region
AGO1-R	AGAACCATCATCCTCATCAACAAG	
CYP-F	AAAGGATGACGTGCTGGGTATGC	Conserve region
CYP-R	AAGGGCGCTGGTGGGTGTAAT	
NF-Y-F	CTTCTCCAGCTACTCGTCTCG	Target site
NF-Y-R	TGGTTCCAATCATGTCTGTTTC	
SCL-F	CAATTCGCTCGGGATCTCAACAT	Conserve region
SCL-R	TTCCGGCGACAAAATTCACACTA	
TCP-F	AAGCAAAAGGGTACAAGAATGG	Conserve region
TCP-R	ATAGAACTGAATGGCGGTGTGG	
FBP-F	TGTGGCCAAGGGAGATT	Target site
FBP-R	GTGAAAGAGAAAAAGAAGGGATACAACA	
GRF-F	GGCCGCCACCGTTCAAGA	Target site
GRF-R	TGGTCGGAGGCATCACAGAGA	

AGO2-F	TCTTTTTTGCCTTCCTCTCTCTCCT	Target site
AGO2-R	CAGACGTAACCTTAATACTTTCTTA	
Germin-F	AGGGTGAGATCTTAGTGGGCTTTGTG	Target site
Germin-R	CAGGCATGGCAGGCTTTGAT	
ITR-F	GTCATGTACTACAGCCCTAC	Target site
ITR-R	AGCAGCGATAGAGAGGAAC	
PFK-F	ATGTGGTGGTCTATGTCCTGGTTTA	Target site
PFK-R	ATGGCCCCCTCGTGAAGT	
FNR-F	GTTCTTTGTTATACAAGGAGGA	
FNR-R	GAACCAGTCAATACCATCT	
